# Docking study, molecular dynamic, synthesis, anti-α-glucosidase assessment, and ADMET prediction of new benzimidazole-Schiff base derivatives

**DOI:** 10.1038/s41598-022-18896-0

**Published:** 2022-09-01

**Authors:** Homa Azizian, Keyvan Pedrood, Ali Moazzam, Yousef Valizadeh, Kimia Khavaninzadeh, Ali Zamani, Maryam Mohammadi-Khanaposhtani, Somayeh Mojtabavi, Mohammad Ali Faramarzi, Samanesadat Hosseini, Yaghoub Sarrafi, Hossein Adibi, Bagher Larijani, Hossein Rastegar, Mohammad Mahdavi

**Affiliations:** 1 Department of Medicinal Chemistry, School of Pharmacy, Iran University of Medical Sciences, Tehran, Iran; 2 Endocrinology and Metabolism Research Center, Endocrinology and Metabolism Clinical Sciences Institute, Tehran University of Medical Sciences, Tehran, Iran; 3 Faculty of Chemistry, University of Mazandaran, Babolsar, Iran; 4 Cellular and Molecular Biology Research Center, Health Research Institute, Babol University of Medical Sciences, Babol, Iran; 5 Department of Pharmaceutical Biotechnology, Faculty of Pharmacy, Tehran University of Medical Sciences, Tehran, Iran; 6 Shahid Beheshti University of Medical Sciences, Tehran, Iran; 7 Cosmetic Products Research Center, Iranian Food and Drug Administration, MOHE, Tehran, Iran

**Keywords:** Biochemistry, Chemical biology

## Abstract

The control of postprandial hyperglycemia is an important target in the treatment of type 2 diabetes mellitus (T2DM). As a result, targeting α-glucosidase as the most important enzyme in the breakdown of carbohydrates to glucose that leads to an increase in postprandial hyperglycemia is one of the treatment processes of T2DM. In the present work, a new class of benzimidazole-Schiff base hybrids **8a–p** has been developed based on the potent reported α-glucosidase inhibitors. These compounds were synthesized by sample recantations, characterized by ^1^H-NMR, ^13^C-NMR, FT-IR, and CHNS elemental analysis, and evaluated against α-glucosidase. All new compounds, with the exception of inactive compound **8g**, showed excellent inhibitory activities (60.1 ± 3.6–287.1 ± 7.4 µM) in comparison to acarbose as the positive control (750.0 ± 10.5). Kinetic study of the most potent compound **8p** showed a competitive type of inhibition (K_i_ value = 60 µM). In silico induced fit docking and molecular dynamics studies were performed to further investigate the interaction, orientation, and conformation of the title new compounds over the active site of α-glucosidase. In silico druglikeness analysis and ADMET prediction of the most potent compounds demonstrated that these compounds were druglikeness and had satisfactory ADMET profile.

## Introduction

Diabetes mellitus (DM) is a primary public health problem stemming from whether deficiency in insulin secretion or decreased insulin sensitivity which results in disturbance of fat, carbohydrate, and protein metabolism^1^. This metabolic disorder that characterized by chronic hyperglycemia is the third highest risk factor for premature mortality according to the 2009 estimation of World Health Organization (WHO), and results to a whole range of serious health problems such as obesity, blindness, excessive urination, enormous appetite, abnormally great thirst, as well as cardiovascular, renal, and neurodegenerative diseases^2,3^. Non-insulin-dependent diabetes mellitus (NIDDM), which is commonly known as T2DM, is the most common type of DM^4^. Unfortunately, T2DM not only affects older adults, but even younger people and children due to the poor diet, lack of exercise, and obesity^5^. Acarbose, as a non-absorbed drug, reduces the glucose level in 25% of the population with impaired glucose tolerant through the modulation of digestion in the intestine^6^. This drug inhibits carbohydrate hydrolyzing enzymes like α-glucosidase and α-amylase. The latter enzymes are responsible for degradation of carbohydrates to glucose and increase postprandial hyperglycemia in T2DM. However, the undesired side effects such as diarrhea, abdominal pain, and nausea are the inevitable consequences of long-term use of acarbose^7–9^. As a result, the need for developing novel α-glucosidase inhibitors is increasing sharply^10,11^.

**Figure 1:**
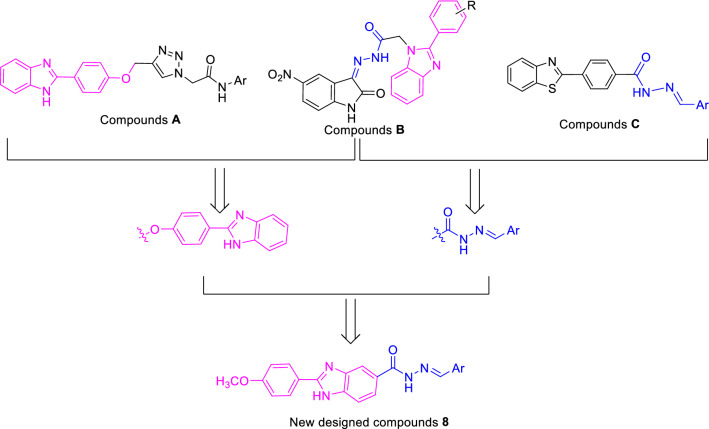
Design strategy for new benzimidazole-Schiff base hybrids **8** as the potent α-glucosidase.

Benzimidazole is a fused heterocycle with benzene and imidazole parts that demonstrated a variety of therapeutic potentials including anti-inflammatory, anticancer, antioxidant, anti-glycation, antimicrobial, β-glucuronidase inhibitor, carbonic anhydrase inhibitor, antiviral, and antiulcer activities^12–15^. Recently, benzimidazole core in the design of the new α-glucosidase inhibitors have been received a lot of attention and the several series of its derivatives were introduced as potent α-glucosidase inhibitors^16–20^. For example, compounds **A** and **B** showed high inhibitory activities against α-glucosidase (Fig. 1). As can be seen in the structure of compounds **B** (Fig. 1), these compounds had a Schiff base moiety in their general scaffold. Schiff base moiety also observed in some of potent α-glucosidase inhibitors such as compounds **C** (Fig. 1)^21–24^. Therefore, our research group decided to combination of benzimidazole and Schiff base moieties for design of benzimidazole-Schiff base hybrids **8** and evaluated them as the new α-glucosidase inhibitors.

## Results and discussion

### Chemistry

**Scheme 1:**
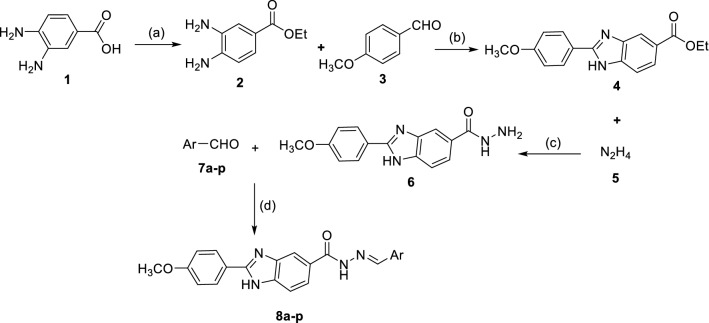
Reagents and conditions for the synthesis of benzimidazole-Schiff base derivatives **8a–p**; (**a**) EtOH, H_2_SO_4_, Reflux, 12 h; (**b**) DMF, Na_2_S_2_O_5_, 100 °C, 5 h; (**c**) EtOH, Room temperature, 16 h; (**d**) PTSA, EtOH, Room temperature, 1 h.

The new benzimidazole-Schiff base derivatives **8a–p** were synthesized through a multistep reaction sequence starting from esterification of 3,4-diaminobenzoic acid **1** in ethanol with catalytic amount of sulfuric acid to provide ethyl 3,4-diaminobenzoate **2**. Next, the latter compound reacted with 4-methoxy benzaldehyde **3** in the presence of Na_2_S_2_O_5_ in DMF at 100 °C to give ethyl 2-(4-methoxyphenyl)-1*H*-benzo[d]imidazole-6-carboxylate **4**. After that, compound **4** reacted with hydrazine hydrate **5** in ethanol at the ambient temperature to give 2-(4-methoxyphenyl)-1*H*-benzo[d]imidazole-6-carbohydrazide **6**. The reaction of the compound **6** with aromatic aldehydes **7a–p** afforded the corresponding final products **8a–p** (Scheme 1). Chemical structures of compounds **8a–p** were elucidated by taking advantage of spectroscopic techniques including ^1^H-NMR, ^13^C-NMR, FT-IR, and CHNS elemental analysis.

### Inhibitory activity of the synthesized compounds 8a–p against α-glucosidase

The newly synthesized benzimidazole-Schiff base derivatives **8a–p** were evaluated against yeast α-glucosidase. The results were listed in Table 1 and revealed that the compounds **8a–p**, with the exception of compound **8d**, with IC_50_ values ≤ 287.1 ± 7.4 μM were significantly more potent than the standard inhibitor acarbose with IC_50_ value of 750.0 ± 10.5 μM. The most active compounds were thiophen-2-yl, 2-fluorophenyl, and phenyl derivatives with IC_50_ values ≤ 70.6 µM (compounds **8p**, **8h**, and **8a**, respectively). Furthermore, compounds **8j**, **8i** and **8k** with 4-fluoro, 3-fluoro, and 2-chloro substituents, respectively, exhibited high anti-α-glucosidase activity (IC_50_ values ≤ 90.0 µM).

**Table 1:** In vitro α-glucosidase inhibitory activities of compounds **8a–p**.


Compound	Ar	IC_50_ (µM)
**8a**	Phenyl	70.6 ± 6.8
**8b**	4-Methylphenyl	101.6 ± 5.6
**8c**	4-Methoxyphenyl	110.0 ± 5.5
**8d**	3,4,5-Trimethoxyphenyl	750 <
**8e**	3-Phenoxyphenyl	127.3 ± 6.5
**8f**	3-Hydroxyphenyl	181.4 ± 8.5
**8g**	4-Hydroxy-3-methoxyphenyl	287.1 ± 7.4
**8h**	2-Fluorophenyl	69.0 ± 4.0
**8i**	3-Fluorophenyl	88.1 ± 6.0
**8j**	4-Fluorophenyl	84.1 ± 4.5
**8k**	2-Chlorophenyl	90.0 ± 7.5
**8l**	4-Chlorophenyl	103.4 ± 5.5
**8m**	3-Bromophenyl	97.0 ± 4.4
**8n**	4-Nitrophenyl	112.4 ± 5.7
**8o**	6-Nitrobenzo[d][1,3]dioxol-5-yl	123.3 ± 6.1
**8p**	Thiophen-2-yl	60.1 ± 3.6
Acarbose	–	750.0 ± 10.5

### Structure–activity relationships (SAR)

As can be seen in Table 1, based on SAR study, activity of compounds **8a–p** against α-glucosidase depended on the aryl group linked to Schiff base moiety. A noteworthy point in the inhibitory activity of these compounds is that the size of the aryl group plays an important role in the observed inhibitory activities. In this regards, the most potent compound was un-substituted compound **8p** with thiophen-2-yl group. Moreover, 2-fluorophenyl derivative **8h** and un-substituted phenyl derivative **8a** were the second and third most potent compounds with IC_50_ values approximately same.

**Scheme 2:**
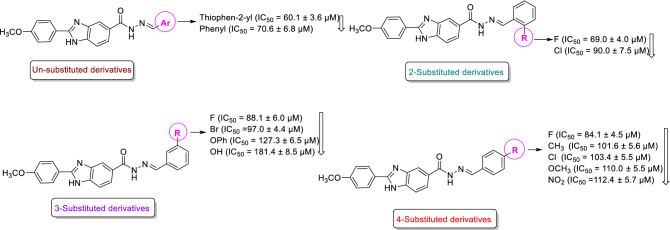
Inhibitory activities of the un-substituted derivatives and derivatives with one substituent on phenyl ring linked to the Schiff base moiety.

The comparison of IC_50_ values of the compounds with substituted phenyl group with one substituent demonstrated that size of substituent had more importance in obtained inhibitory activities in comparison to nature of substituent, with one exception which will be explained later. As can be seen in Scheme 2, in 2, 3, or 4-substituted phenyl derivatives, compounds with smaller substitutions are more effective, with the exception of 3-hydroxy derivative **8f** that was less effective that 3-phenoxyphenyl derivative **8e**. In this regards, the order of inhibitory activities in 2-substituted phenyl derivatives was F (compound **8h**) > Cl (compound **8k**) and the order of inhibitory activities in 4-substituted phenyl derivatives was F (compound **8j)** > CH_3_ (compound **8b**) > Cl (compound **8l**) > OCH_3_ (compound **8c**) > NO_2_ (compound **8n**). Furthermore, among the 3-substituted phenyl derivatives, fluoro derivative (compound **8i**) was more potent than bromo derivative (compound **8m**).

**Scheme 3:**
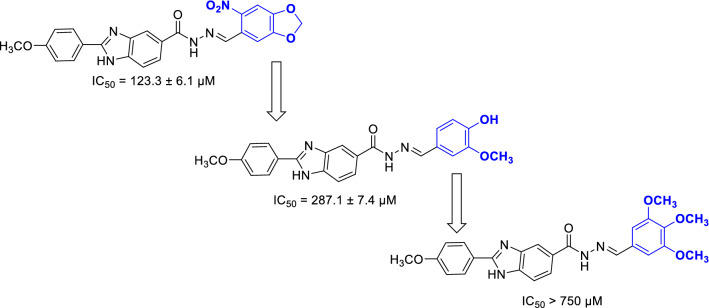
Inhibitory activities of the derivatives with two or three substituents on phenyl ring linked to the Schiff base moiety.

The observed IC_50_ values of the newly synthesized compounds with two or three substituents on phenyl ring demonstrated that 6-nitrobenzo[d][1,3]dioxol-5-yl derivative **8o** was more potent than 4-hydroxy-3-methoxyphenyl derivative **8g** and 3,4,5-trimethoxyphenyl derivative **8d** was inactive. It is worthy to note that 6-nitrobenzo[d][1,3]dioxol-5-yl derivative **8o** was also more potent than 3-hydroxy phenyl derivative **8f**. The comparison of the compound **8o** with other compounds with one substituent on phenyl ring demonstrated that inhibitory of this compound is near to 3-phenoxyphenyl derivative **8e** and weaker than remaining one-substituted derivatives (Scheme 3).

### Enzyme kinetic study

**Figure 2:**
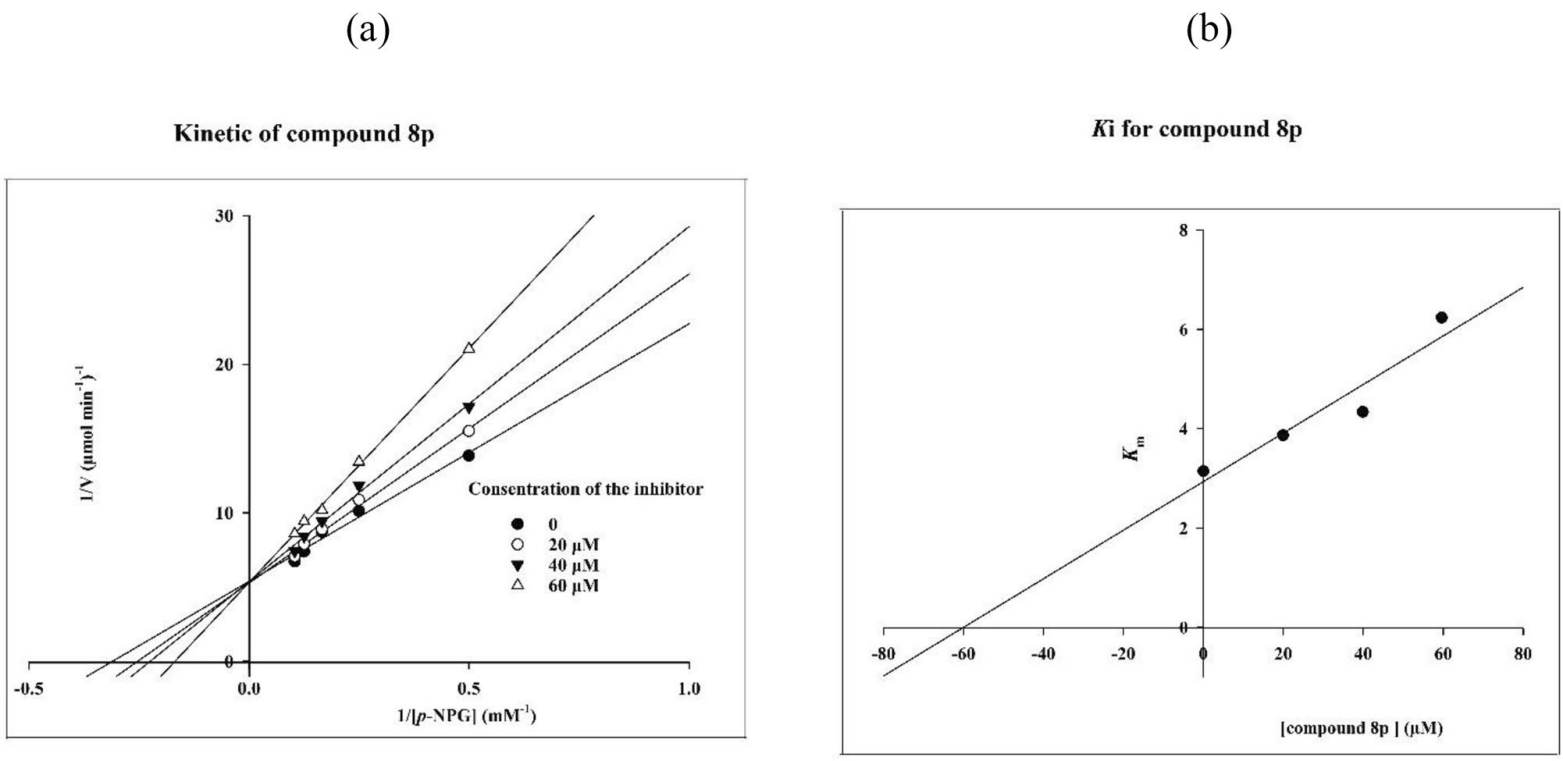
Kinetics of α-glucosidase inhibition by compound **8p**. (**a**) The Lineweaver– Burk plot in the absence and presence of different concentrations of compound **8p**; (**b**) the secondary plot between *K*_m_ and various concentrations of compound **8p**.

To obtain the inhibition mode of the new synthesized compounds against α-glucosidase, the enzyme kinetic study of the most active compound **8p** was performed. According to Fig. 2a, the Lineweaver–Burk plot showed that with increasing concentration of compound **8p**, the *K*_m_ gradually increased while *V*_*max*_ remained unchanged. Therefore, compound **8p** compete with the substrate for binding to the active site and is a competitive inhibitor. Furthermore, the plot of the *K*_m_ versus different concentrations of compound **8p** gave an estimate of K_i_ as the inhibition constant (Fig. 2b). K_i_ value for the latter compound was 60 µM.

### Docking study

The validated docking method was then used to analysis of the binding modes of the newly synthetized compounds **8a**–**p** over the α-glucosidase active site in comparison to acarbose as a standard inhibitor of this enzyme. The reliability of the induced fit docking procedure was conducted according to our previous set up studies based on re-docking of α-d-glucose as the enzyme substrate^25,26^.

**Figure 3:**
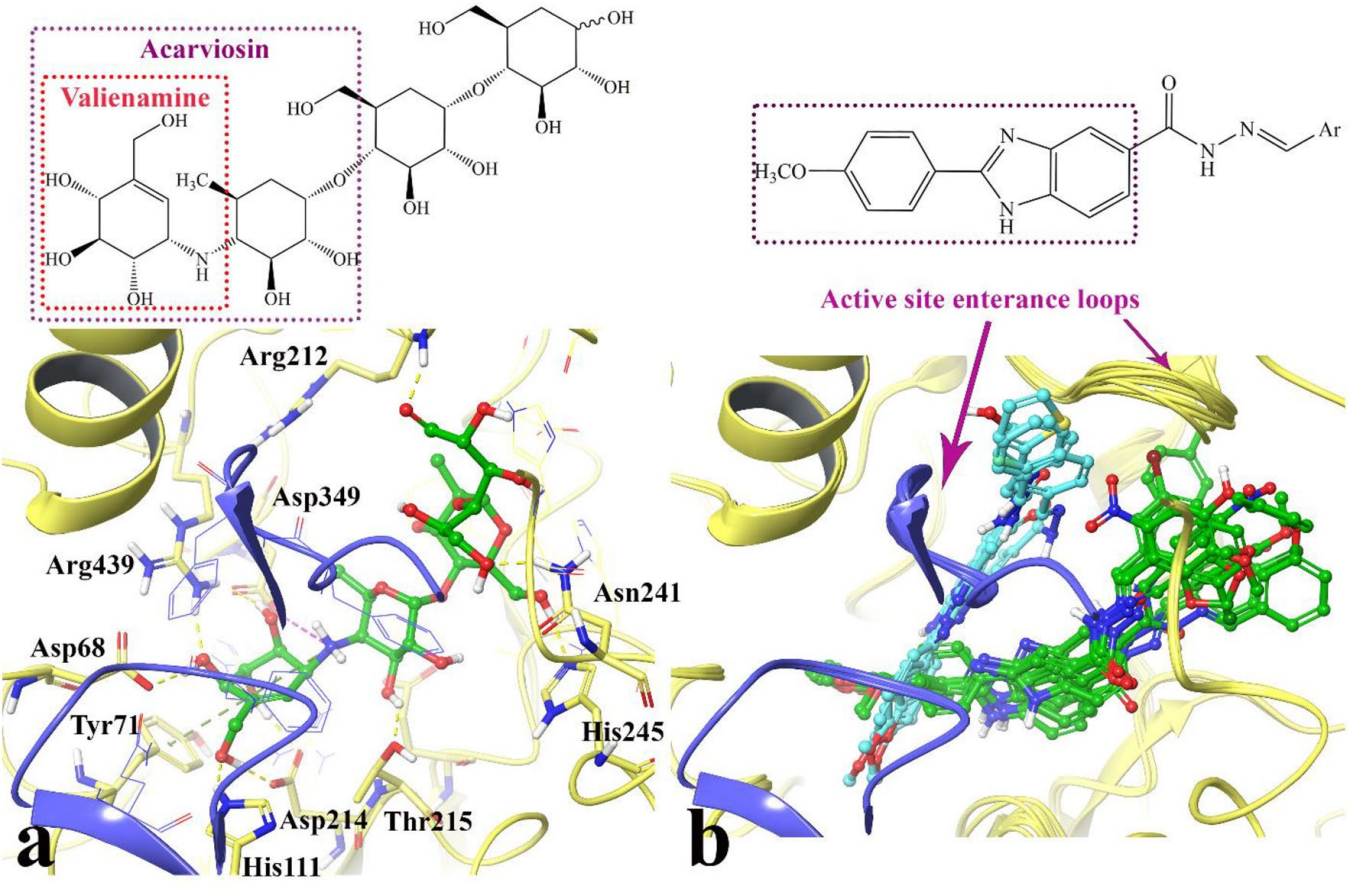
Induced fit docked representation of acarbose (**a**) and the superimposed of the synthesized compounds (**b**). The N-terminal domain and the subdomain of the α-glycosidase are colored in yellow and blue, respectively. Compounds **8p**, **8a**, **8h**, and **8f** with lower steric size shown in cyan color while compounds **8d**, **8g**, **8e**, **8c**, **8b**, **8m**, **8k**, **8n**, **8l**, and **8o** with higher steric size shown in green color.

α-Glucosidase in complex with acarbose was showed in Fig. 3a. The valienamine moiety which is corresponds to the non-reducing terminal of acarbose interacted with Asp68, Tyr71, His111, Asp214, Asp349 and His348 over the − 1 and + 1 subsides at the bottom of the active site also Thr215 formed H-bond with the 6-deoxyglucose subunit at the acarviosine moiety. Furthermore, the reducing terminal of acarbose formed H-bond interaction with Asn241 and Arg312 (with 1.81 Å and 1.76 Å) at the + 2 and + 3 subsides, respectively.

Figure 3b represents superimposed orientation of the best conformational pose and energy valued docked complex of the compounds. It depicts that the synthesized compounds have two different fitting-in conformers inside the active site.

The first one includes compounds **8p**, **8a**, **8h**, and **8f** which are colored in cyan. The orientation of the mentioned compounds is in the way that their low steric substituted benzylidene hydrazide moiety pointed inside the mouth of the active site and stacked between the two loops at the large hydrophobic entrance of the active site; one from N-terminal domain A (in yellow) and the other one from subdomain B (in blue). Also, the second conformation is belonging to compounds **8d**, **8g**, **8e**, **8c**, **8b**, **8m**, **8k**, **8n**, **8l**, and **8o** which are colored in green. The orientation of these compounds is in the way that the bulkier substituted at benzylidene hydrazide moiety turned toward the proximal part of the active site with large hydrophobic space.

Moreover, similar to acarviosin moiety of acarbose, the 4-methoxy phenyl benzimidazole moiety of the both of the mentioned orientation pointed toward the − 1 and + 1 subsides. Based on the observed result, it can be concluded that the same positioning of 4-methoxy phenyl benzimidazole moiety as acarbose into the − 1 and + 1 subsides is important to reserve high enzyme inhibition activity as observed in almost all of the compounds. So, it can propose that the 4-methoxy phenyl benzimidazole moiety of the newly synthesized compound and the acarviosin unit of acarbose have the same role in the enzyme inhibition process.

In addition, the higher inhibitory activity among these compounds is controlled efficiently by the size of the substitution over benzylidene hydrazide moiety in which smaller size substitutions can accommodate inside the active site entrance consequently inhibit more efficiently the enzyme activity (as can be seen by compounds **8p**, **8a**, **8h**, and **8f**).

### Molecular dynamics

The molecular dynamics (MD) simulation performed in order to confirm the compound stability over the enzyme active site. For this purpose, the structural deviation experience by the most potent compound (compounds **8p**) have been studied over the active pocket cavity.

**Figure 4:**
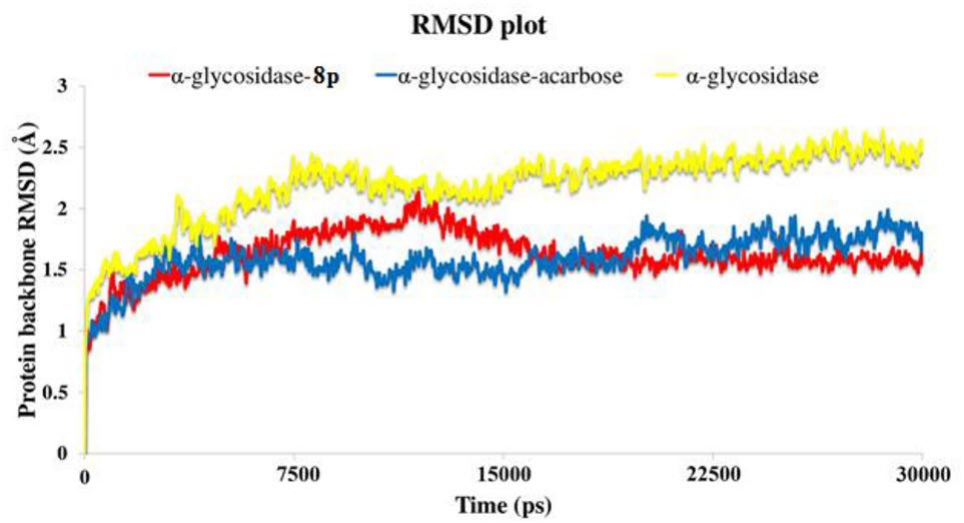
RMSD of the α-glycosidase backbone in complexed with acarbose (in blue), compound **8p** (in red) and the unbound enzyme (in yellow) for over 30 ns MD simulation time.

The root mean square deviation (RMSD) of the enzyme’s backbone was analyzed over during 30 ns MD simulation in order to study the perturbation of the protein–ligand complex. The RMSD value of the unbounded α-glycosidase enzyme depicts higher value than the value of the enzymes complexe with compound **8p** and acarbose (Fig. 4). The unbounded enzyme RMSD value significantly increased during the first 7.5 ns up to 2.3 Å and fluctuated until 15 ns and become more stable for the last 5 ns of the simulation time with the value of 2.5 Å (Fig. 4, yellow line). Moreover, based on the RMSD value of α-glycosidase complexed with acarbose and compound **8p**, the bounded-state enzymes were stable during the simulation time with the lower RMSD value of 1.6 Å and 1.7 Å, respectively (Fig. 4, blue and red line) which shows that ligand-active site bound-state has significant impact on α-glycosidase structural stability. The mentioned result indicates that the employed simulation time has been enough to obtain an equilibrium structure over the simulation time.

**Figure 5:**
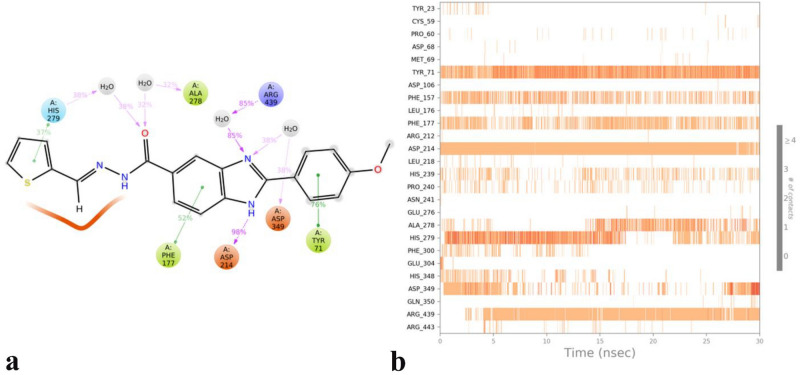
The timeline representation of the interactions shows the residues interact with compound **8p** in each trajectory frame (more than one specific contact with the ligand is represented by a darker shade of orange) (**a**). The simulation interactions diagram panel in which the stacked bar charts are normalized over the course of the trajectory: some protein residues may make multiple contacts with the ligand (**b**) (Desmond v5.3).

Based on the MD investigation of compound **8p**, it reveals that the terminal 4-methoxy phenyl group interacted with Tyr71 at the bottom of the active site for almost the whole simulation time (Fig. 5). Also, the benzimidazole ring has an important role in stabilizing **8p** over the + 1 subside by forming stable H-bond, H-bond water-mediated and π–π hydrophobic interaction with Asp214 (conserve acidic residue) and Arg439 and Phe177 for 98%, 85% and 52% of simulation time, respectively.

Additionally, compound **8p** stabilized over the + 2 subside through water mediated H-bond interaction of Schiff base group with Ala278 and His279 and an extra π-π hydrophobic interaction with His279 for more than one third of the simulation time.

The mentioned result show that the conformation and the correspond non- bonding interaction of compound **8p** were stable during the whole simulation time in which provide a reliable interpretation for the observed interaction over the α-glycosidase active site.

### In silico druglikeness, ADME, and toxicity studies

In silico druglikeness/ADME/T properties of the most potent compounds **8a**, **8h**, **8p**, and positive control acarbose were calculated using PreADMET as an online software and the obtained results were listed in Table 2.^27^ As can be seen in Table 2, the new compounds **8a**, **8h**, and **8p** followed of Lipinski 'Rule of five' while acarbose did not follow in this rule. All the studied compounds have poor permeability to Caco-2 cell and skin. Compound **8p** and acarbose are in normal rang for permeability to blood brain barrier (BBB) while compounds **8a** and **8h** have poor permeability to BBB. Furthermore, compounds **8a**, **8h**, and **8p** have high human intestinal absorption (HIA) while acarbose did not have HIA. Predicting the toxicity of the title compounds **8a**, **8h**, **8p**, and acarbose by PreADMET toxicity server demonstrated that all these compounds are mutagen (Ames test). In term of cardiotoxicity (hERG inhibition), compound **8a** had high risk while compounds **8h** and **8p** had medium risk. Cardiotoxicity of acarbose was ambiguous. Our new compounds **8a**, **8h**, and **8p** did not have carcinogenic effect on mouse while acarbose had carcinogenic effect on mouse. Moreover, compounds **8a**, **8p**, and acarbose did not have carcinogenic effect on rat while compound **8h** presumably is a carcinogen agent for rat.

**Table 2:** Druglikeness/ADME/T profile of the most potent compounds **8a**, **8h**, **8p**, and standard drug acarbose.

ADME/T^a^	Compound			
	8a	8h	8p	Acarbose
Rule of five	Suitable	Suitable	Suitable	Suitable
Caco2	9.77925	10.7172	13.3575	9.44448
HIA	92.711389	92.728877	92.754226	0.000000
BBB	1.23514	1.42931	0.308469	0.0271005
Skin permeability	− 3.50077	− 3.73046	− 3.9956	− 5.17615
Ames test	Mutagen	Mutagen	Mutagen	Mutagen
hERG inhibition	High risk	Medium risk	Medium risk	Ambiguous
Carcino Mouse	Negative	Negative	Negative	Positive
Carcin Rat	Negative	Positive	Negative	Negative

^a^The recommended ranges for Caco2: < 25 poor, > 500 great, HIA: > 80% is high < 25% is poor, BBB = − 3.0 to 1.2, and Skin_Permeability = − 8.0 to − 1.0.

### In vitro cytotoxicity assay

In order to further evaluate on the toxicity of the synthesized compounds, cytotoxicity of the most potent compounds **8a**, **8h**, and **8p** was determined by MTT assay on HDF and MCF-7 as human normal and cancer cell lines, respectively^28^. Obtained results revealed that these compounds at 200 μM were non-cytotoxic against studied cell lines.

## Conclusion

In conclusion, new hybrids of benzimidazole and Schiff base derivatives, compounds **8a–p**, were synthesized and their inhibitory effects were evaluated against α-glucosidase. These newly synthesized compounds exhibited excellent α-glucosidase inhibitory activity in comparison with acarbose as the positive control of assay. In this regards, the most potent compounds were compounds **8p**, **8h**, and **8a** with thiophene, phenyl, and 2-fluoro phenyl in Schiff base moiety and only a compound (compound **8g**) with 3,4,5-trimethoxy phenyl in the latter moiety was inactive against α-glucosidase. The SAR study revealed that the size of aryl group of Schiff base moiety plays an important role in the obtained inhibition effects. Based on SAR study, compounds containing un-substituted aryl group or aryl group with small substituents had more inhibitory activity in comparison to compounds with bulky aryl groups or aryl group with bigger substituents. IFD and MD studies showed that the stable positioning of benzimidazole moiety of the scaffold of the newly synthesized compound into the active site is the same functionality as acarviosin unit of acarbose which may have an important role to reserve high enzyme inhibition activity. In addition, the higher inhibition activity of compounds **8p**, **8a**, and **8h** compounds is controlled efficiently by the size of the substitution over aryl group of Schiff base moiety in which smaller size substitutions at this section can accommodate inside the active site entrance consequently inhibit more efficiently the enzyme activity. Furthermore, it was predicted that the latter compounds were druglikeness and had a good profile in term of ADMET.

## Experimental

### Synthesis of ethyl 3,4-diaminobenzoate 2

The 3,4-diaminobenzoic acid **1** (5 mmol) was poured in dry ethanol (20 ml), and H_2_SO_4_ was added to the medium and the obtained mixture was refluxed for 12 h. Then, formed green solid was filtered off after pouring the mixture into water to give ethyl 3,4-diaminobenzoate **2**.

### Synthesis of ethyl 2-(4-methoxyphenyl)-1H-benzo[d]imidazole-5-carboxylate 4

A mixture of ethyl 3,4-diaminobenzoate **2** (5 mmol), 4-methoxybenzaldehyde **3** (5 mmol), and Na_2_S_2_O_5_ (5.5 mmol) in DMF (20 mL) was stirred at 100 °C for 5 h at the closed condition. Then, the mixture was poured in the cold water and the pure 2-(4-methoxyphenyl)-1*H*-benzo[d]imidazole-5-carboxylate **4** was filtered off.

### Synthesis of 2-(4-methoxyphenyl)-1H-benzo[d]imidazole-5-carbohydrazide 6

The mixture of ethyl 2-(4-methoxyphenyl)-1*H*-benzo[d]imidazole-5-carboxylate **4** (5 mmol) and hydrazine **5** (15 ml) was stirred in ethanol (20 ml) at the ambient temperature for 16 h. After completion of the reaction (monitored by the TLC), the participated product **6** was filtrated and purified by recrystallization in ethyl acetate.

### General procedure for the synthesis of carbohydrazide-benzimidazole derivatives 8a–p

A mixture of 2-(4-methoxyphenyl)-1*H*-benzo[d]imidazole-5-carbohydrazide **6** (1 mmol) and appropriate benzaldehydes **7a–p** (1 mmol) in the presence of a catalytic amount of *para*-toluenesulfonic acid (PTSA) in ethanol was stirred at room temperature for 1 h. Then, the mixture was added to cold water, and the precipitates were collected by filtration and recrystallized in ethanol to obtain the corresponding final products **8a–p**.

### (*E*)-*N'*-benzylidene-2-(4-methoxyphenyl)-1H-benzo[d]imidazole-5-carbohydrazide (8a)

Yield 72% (266 mg), white solid: m.p. > 250 °C. IR (KBr) υ: 3225, 1667, 1628, 1591, 1306, 1210. ^1^H NMR (300 MHz, DMSO-*d*_6_) δ 12.12 (s, 1H), 8.54 (s, 1H), 8.41 (s, 1H), 8.28 (s, 1H), 8.22 (d, *J* = 8.7 Hz, 2H), 8.08 (d, *J* = 8.7 Hz, 1H), 7.90 (d, *J* = 8.6 Hz, 1H), 7.78–7.70 (m, 1H), 7.47 (t, *J* = 3.3 Hz, 2H), 7.28 (d, *J* = 8.6 Hz, 2H), 7.15 (d, *J* = 7.8 Hz, 2H), 3.90 (s, 3H). ^13^C NMR (75 MHz, DMSO) δ 163.93, 162.86, 151.40, 148.78, 145.29, 138.76, 134.72, 134.61, 132.34, 131.07, 130.65, 129.32, 128.77, 127.61, 125.99, 115.74, 115.42, 114.18, 56.33. Anal.Calcd for C_22_H_18_N_4_O_2_: C 71.34, H 4.90, N 15.13; Found: C 71.46, H 4.75, N 15.26.

### (*E*)-2-(4-methoxyphenyl)-*N'*-(4-methylbenzylidene)-1H-benzo[d]imidazole-5-carbohydrazide (8b)

Yield 69% (264 mg), white solid: m.p. > 250 °C. IR (KBr) υ: 3255, 1656, 1625, 1586, 1276, 1080. ^1^H NMR (300 MHz, DMSO-*d*_6_) δ 12.02 (s, 1H), 8.47 (s, 1H), 8.26 (s, 1H), 8.20 (d, *J* = 8.7 Hz, 2H), 8.07 (d, *J* = 8.6 Hz, 1H), 7.87 (d, *J* = 8.6 Hz, 1H), 7.61 (t, *J* = 7.8 Hz, 3H), 7.24 (d, *J* = 8.4 Hz, 2H), 7.15 (d, *J* = 7.9 Hz, 2H), 3.88 (s, 3H), 2.28 (s, 3H). ^13^C NMR (75 MHz, DMSO) δ 163.92, 162.74, 151.21, 148.74, 145.11, 140.46, 138.95, 134.45, 132.00, 130.63, 129.88, 128.85, 127.54, 126.03, 115.71, 115.30, 114.05, 56.29, 21.27. Anal.Calcd for C_23_H_20_N_4_O_2_: C 71.86, H 5.24, N 14.57; Found: C 71.71, H 5.42, N 14.36.

### (*E*)-*N'*-(4-methoxybenzylidene)-2-(4-methoxyphenyl)-1H-benzo[d]imidazole-5-carbohydrazide (8c)

Yield 75% (300 mg), white solid: m.p. > 250 °C. IR (KBr) υ: 3236, 1670, 1621, 1602, 1281, 1110. ^1^H NMR (300 MHz, DMSO-*d*_6_) δ 11.90 (s, 1H), 8.43 (s, 1H), 8.33 (s, 1H), 8.23 (s, 1H), 8.16 (d, *J* = 8.5 Hz, 2H), 8.04 (d, *J* = 8.7 Hz, 1H), 7.82 (d, *J* = 8.5 Hz, 1H), 7.67 (d, *J* = 7.8 Hz, 1H), 7.57 (s, 1H), 7.16 (d, *J* = 8.0 Hz, 2H), 6.94 (d, *J* = 8.4 Hz, 2H), 3.83 (s, 3H), 3.75 (s, 3H). ^13^C NMR (75 MHz, DMSO) δ 163.89, 162.51, 161.28, 151.02, 148.66, 144.85, 139.12, 134.20, 132.00, 131.13, 130.57, 129.14, 128.90, 127.19, 126.03, 115.60, 114.97, 114.66, 114.00, 56.20, 55.67. Anal.Calcd for C_23_H_20_N_4_O_3_: C 68.99, H 5.03, N 13.99; Found: C 68.69, H 5.21, N 14.14.

### (*E*)-2-(4-methoxyphenyl)-*N'*-(3,4,5-trimethoxybenzylidene)-1H-benzo[d]imidazole-5-carbohydrazide (8d)

Yield 71% (326 mg), white solid: m.p. > 250 °C. IR (KBr) υ: 3222, 1676, 1629, 1593, 1269, 1195, 1040. ^1^H NMR (300 MHz, DMSO-*d*_6_) δ 12.11 (s, 1H), 8.47 (s, 1H), 8.37 (s, 1H), 8.28 (d, *J* = 1.4 Hz, 1H), 8.22 (d, *J* = 8.9 Hz, 2H), 8.08 (dd, *J* = 8.6, 1.5 Hz, 1H), 7.90 (d, *J* = 8.6 Hz, 1H), 7.59 (d, *J* = 8.1 Hz, 2H), 7.28 (d, *J* = 8.7 Hz, 2H), 7.15 (d, *J* = 7.9 Hz, 2H), 7.04 (s, 2H), 3.91 (s, 3H), 3.85 (s, 3H), 3.73 (s, 3H). ^13^C NMR (75 MHz, DMSO) δ 163.94, 162.83, 153.65, 151.37, 148.80, 145.29, 139.77, 138.75, 134.58, 132.31, 131.11, 130.63, 130.24, 128.76, 125.98, 115.73, 115.41, 114.03, 104.80, 60.60, 56.40, 56.32. Anal.Calcd for C_23_H_20_N_4_O_3_: C 65.21, H 5.25, N 12.17; Found: C 65.43, H 5.49, N 12.01.

### (*E*)-2-(4-methoxyphenyl)-*N'*-(3-phenoxybenzylidene)-1H-benzo[d]imidazole-5-carbohydrazide (8e)

Yield 74% (341 mg), white solid: m.p. > 250 °C. IR (KBr) υ: 3269, 1669, 1631, 1587, 1496, 1302, 1233, 1186. ^1^H NMR (300 MHz, DMSO-*d*_6_) δ 12.13 (s, 1H), 8.52 (s, 1H), 8.38 (s, 1H), 8.26 (s, 1H), 8.22 (d, *J* = 8.6 Hz, 2H), 8.06 (d, *J* = 8.6 Hz, 1H), 7.88 (d, *J* = 8.5 Hz, 1H), 7.54–6.93 (m, 11H), 3.90 (s, 3H). ^13^C NMR (75 MHz, DMSO) δ 163.90, 162.85, 157.75, 156.75, 151.42, 148.02, 145.27, 138.78, 136.75, 134.72, 132.41, 131.05, 130.66, 130.62, 128.77, 126.00, 125.47, 124.33, 123.31, 120.79, 119.45, 116.11, 115.72, 115.48, 114.17, 56.30. Anal.Calcd for C_28_H_22_N_4_O_3_: C 72.71, H 4.79, N 12.11; Found: C 72.69, H 4.56, N 12.32.

### (*E*)-*N'*-(3-hydroxybenzylidene)-2-(4-methoxyphenyl)-1H-benzo[d]imidazole-5-carbohydrazide (8f)

Yield 76% (293 mg), white solid: m.p. > 250 °C. IR (KBr) υ: 3540, 3102, 1671, 1623, 1572, 1221, 1105. ^1^H NMR (300 MHz, DMSO-*d*_6_) δ 12.05 (s, 1H), 8.46 (s, 1H), 8.34 (s, 1H), 8.26 (s, 1H), 8.19 (d, *J* = 8.5 Hz, 2H), 8.06 (d, *J* = 8.7 Hz, 1H), 7.86 (d, *J* = 8.6 Hz, 1H), 7.37–6.97 (m, 6H), 6.92–6.74 (m, 1H), 3.87 (s, 3H). ^13^C NMR (75 MHz, DMSO) δ 163.67, 162.33, 161.58, 150.89, 146.79, 145.29, 141.23, 139.10, 132.40, 130.29, 128.56, 125.97, 124.62, 121.04, 118.12, 115.71, 115.65, 114.48, 114.39, 56.26. Anal.Calcd for C_22_H_18_N_4_O_3_: C 68.38, H 4.70, N 14.50; Found: C 68.59, H 4.62, N 14.29.

### (*E*)-*N'*-(4-hydroxy-3-methoxybenzylidene)-2-(4-methoxyphenyl)-1H-benzo[d]imidazole-5-carbohydrazide (8g)

Yield 70% (291 mg), white solid: m.p. > 250 °C. IR (KBr) υ: 3542, 3048, 1671, 1633, 1592, 1200, 1100. ^1^H NMR (300 MHz, DMSO-*d*_6_) δ 11.95 (s, 1H), 8.43 (s, 1H), 8.38 (s, 1H), 8.26 (d, *J* = 1.4 Hz, 1H), 8.20 (d, *J* = 8.7 Hz, 2H), 8.06 (dd, *J* = 8.6, 1.5 Hz, 1H), 7.87 (d, *J* = 8.5 Hz, 1H), 7.33 (s, 1H), 7.24 (d, *J* = 8.8 Hz, 1H), 7.15 (d, *J* = 7.9 Hz, 2H), 7.10 (dd, *J* = 8.3, 1.8 Hz, 1H), 6.88 (d, *J* = 8.1 Hz, 1H), 3.88 (s, 3H), 3.83 (s, 3H). ^13^C NMR (75 MHz, DMSO) δ 163.89, 162.64, 151.26, 149.60, 149.42, 148.50, 145.05, 138.93, 134.46, 132.28, 131.21, 130.60, 128.83, 126.10, 125.99, 122.75, 115.92, 115.68, 115.33, 113.97, 109.45, 56.27, 55.98. Anal.Calcd for C_23_H_20_N_4_O_4_: C 66.34, H 4.84, N 13.45; Found: C 66.16, H 4.62, N 13.36.

### (*E*)-*N'*-(2-fluorobenzylidene)-2-(4-methoxyphenyl)-1H-benzo[d]imidazole-5-carbohydrazide (8h)

Yield 79% (303 mg), white solid: m.p. 267‒269 °C. IR (KBr) υ: 3261, 1662, 1610, 1578, 1250, 1130, 1016. ^1^H NMR (300 MHz, DMSO-*d*_6_) δ 12.20 (s, 1H), 8.74 (s, 1H), 8.47 (s, 1H), 8.26 (s, 1H), 8.20 (d, *J* = 8.5 Hz, 2H), 8.07 (d, *J* = 8.6 Hz, 1H), 8.00–7.80 (m, 2H), 7.48 (t, *J* = 7.4 Hz, 1H), 7.35–7.20 (m, 2H), 7.15 (d, *J* = 7.8 Hz, 2H), 3.88 (s, 3H). ^13^C NMR (75 MHz, DMSO) δ 163.89, 162.91 (d, ^1^*J*_CF_ = 242.25 Hz), 162.83, 151.37, 147.32, 145.23 (d, ^3^*J*_CF_ = 7.5 Hz), 138.83, 134.63, 132.58 (d, ^3^*J*_CF_ = 7.5 Hz), 132.30, 130.81, 130.52 (d, ^3^*J*_CF_ = 8.25 Hz), 128.77, 125.98, 125.52, 124.12 (d, ^4^*J*_CF_ = 2.25 Hz), 117.22 (d, ^2^*J*_CF_ = 16.5 Hz), 115.71, 115.68, 114.23 (d, ^2^*J*_CF_ = 15 Hz),, 114.15, 56.27. Anal.Calcd for C_22_H_17_FN_4_O_2_: C 68.03, H 4.41, N 14.43; Found: C 68.28, H 4.31, N 14.63.

### (*E*)-*N'*-(3-fluorobenzylidene)-2-(4-methoxyphenyl)-1H-benzo[d]imidazole-5-carbohydrazide (8i)

Yield 78% (302 mg), white solid: m.p. > 250 °C. IR (KBr) υ: 3261, 1662, 1626, 1578, 1256, 1133, 1016. ^1^H NMR (300 MHz, DMSO-*d*_6_) δ 12.20 (s, 1H), 8.52 (s, 1H), 8.40 (s, 1H), 8.26 (s, 1H), 8.21 (d, *J* = 8.9 Hz, 1H), 8.07 (d, *J* = 8.3 Hz, 1H), 7.89 (d, *J* = 8.6 Hz, 1H), 7.59 (d, *J* = 8.1 Hz, 1H), 7.56–7.44 (m, 2H), 7.26 (d, *J* = 8.6 Hz, 2H), 7.15 (d, *J* = 7.9 Hz, 2H), 3.89 (s, 3H). ^13^C NMR (75 MHz, DMSO) δ 164.47 (d, ^1^*J*_CF_ = 242.25 Hz), 163.93, 162.89, 151.41, 147.32, 145.22, 138.81, 137.33 (d, ^3^*J*_CF_ = 7.5 Hz), 134.65, 132.31, 131.44 (d, ^3^*J*_CF_ = 8.25 Hz), 130.81, 130.64, 128.78, 125.99, 125.53, 124.04 (d, ^4^*J*_CF_ = 2.25 Hz), 117.47 (d, ^2^*J*_CF_ = 20.25 Hz), 115.71, 115.36, 114.15, 113.54 (d, ^2^*J*_CF_ = 22.5 Hz), 56.30. Anal.Calcd for C_22_H_17_FN_4_O_2_: C 68.12, H 4.38, N 14.53; Found: C 68.33, H 4.48, N 14.63.

### (*E*)-*N'*-(4-fluorobenzylidene)-2-(4-methoxyphenyl)-1H-benzo[d]imidazole-5-carbohydrazide (8j)

Yield 80% (310 mg), white solid: m.p. > 250 °C. IR (KBr) υ: 3259, 1656, 1616, 1589, 1245, 1150, 1004. ^1^H NMR (300 MHz, DMSO-*d*_6_) δ 12.13 (s, 1H), 8.53 (s, 1H), 8.23 (s, 1H), 8.28 (s, 1H), 8.22 (d, *J* = 8.9 Hz, 2H), 8.08 (d, *J* = 8.6 Hz, 1H), 7.91 (d, *J* = 8.6 Hz, 1H), 7.82 (dd, *J* = 8.5, 5.6 Hz, 2H), 7.39–7.24 (m, 2H), 7.14 (d, *J* = 7.9 Hz, 2H), 3.92 (s, 3H). ^13^C NMR (75 MHz, DMSO) δ 165.29 (d, ^1^*J*_CF_ = 246 Hz), 163.87, 162.90, 151.57, 147.57, 145.53, 138.58, 134.93, 132.65, 131.37 (d, ^4^*J*_CF_ = 2.25 Hz), 130.61, 129.84 (d, ^3^*J*_CF_ = 8.25 Hz), 128.70, 125.98, 116.56, 116.27, 115.75, 114.22 (d, ^2^*J*_CF_ = 10.5 Hz), 56.33. Anal.Calcd for C_22_H_17_FN_4_O_2_: C 68.03, H 4.41, N 14.43; Found: C 67.92, H 4.39, N 14.51.

### (*E*)-*N'*-(2-chlorobenzylidene)-2-(4-methoxyphenyl)-1H-benzo[d]imidazole-5-carbohydrazide (8k)

Yield 77% (311 mg), white solid: m.p. > 250 °C. IR (KBr) υ: 3249, 1664, 1622, 1591, 1568, 1322, 1226, 1001, 768. ^1^H NMR (300 MHz, DMSO-*d*_6_) δ 12.30 (s, 1H), 8.90 (s, 1H), 8.43 (s, 1H), 8.27 (d, *J* = 1.5 Hz, 1H), 8.21 (d, *J* = 8.9 Hz, 2H), 8.13–7.95 (m, 2H), 7.90 (d, *J* = 8.6 Hz, 1H), 7.55–7.37 (m, 3H), 7.15 (d, *J* = 7.9 Hz, 2H), 3.89 (s, 3H). ^13^C NMR (75 MHz, DMSO) δ 163.91, 162.88, 151.43, 145.22, 144.51, 138.80, 134.72, 133.74, 132.37, 131.99, 130.64, 130.38, 128.77, 128.04, 127.33, 126.01, 115.69, 115.39, 114.19, 56.29. Anal.Calcd for C_22_H_17_ClN_4_O_2_: C 65.27, H 4.23, N 13.84; Found: C 65.16, H 4.46, N 13.64.

### (*E*)-*N'*-(4-chlorobenzylidene)-2-(4-methoxyphenyl)-1H-benzo[d]imidazole-5-carbohydrazide (8l)

Yield 79% (319 mg), white solid: m.p. > 250 °C. IR (KBr) υ: 3231, 1656, 1620, 1596, 1242, 1113, 726. ^1^H NMR (300 MHz, DMSO-*d*_6_) δ 12.17 (s, 1H), 8.52 (s, 1H), 8.38 (s, 1H), 8.28 (d, *J* = 1.5 Hz, 1H), 8.22 (d, *J* = 8.9 Hz, 2H), 8.07 (d, *J* = 8.6 Hz, 1H), 7.91 (d, *J* = 8.6 Hz, 1H), 7.78 (d, *J* = 8.2 Hz, 2H), 7.31 (d, *J* = 8.2 Hz, 2H), 7.14 (d, *J* = 7.9 Hz, 2H), 3.92 (s, 3H). ^13^C NMR (75 MHz, DMSO) δ 163.82, 162.99, 151.79, 147.29, 145.36, 138.34, 137.32, 135.08, 133.70, 130.56, 129.45, 129.22, 128.67, 125.98, 116.12, 115.73, 114.21, 56.33. Anal.Calcd for C_22_H_17_ClN_4_O_2_: C 65.27, H 4.23, N 13.84; Found: C 65.51, H 4.43, N 13.61.

### (*E*)-*N'*-(3-bromobenzylidene)-2-(4-methoxyphenyl)-1H-benzo[d]imidazole-5-carbohydrazide (8m)

Yield 69% (309 mg), white solid: m.p. > 250 °C. IR (KBr) υ: 3241, 1661, 1627, 1588, 1221, 1010, 687. ^1^H NMR (300 MHz, DMSO-*d*_6_) δ 12.19 (s, 1H), 8.49 (s, 1H), 8.39 (s, 1H), 8.25 (s, 1H), 8.20 (d, *J* = 8.6 Hz, 2H), 8.06 (d, *J* = 8.7 Hz, 1H), 7.88 (d, *J* = 8.6 Hz, 1H), 7.70–7.60 (m, 2H), 7.47 (d, *J* = 4.5 Hz, 2H), 7.15 (d, *J* = 7.8 Hz, 2H), 3.89 (s, 3H). ^13^C NMR (75 MHz, DMSO) δ 163.89, 162.83, 151.38, 146.95, 145.20, 138.84, 136.94, 134.67, 134.10, 132.33, 131.17, 130.61, 128.80, 126.67, 126.32, 126.00, 115.68, 115.37, 114.14, 56.28. Anal.Calcd for C_22_H_17_BrN_4_O_2_: C 58.81, H 3.81, N 12.47; Found: C 58.92, H 3.68, N 12.35.

### (*E*)-2-(4-methoxyphenyl)-*N'*-(4-nitrobenzylidene)-1H-benzo[d]imidazole-5-carbohydrazide (8n)

Yield 85% (352 mg), light − brown solid: m.p. > 250 °C. IR (KBr) υ: 3256, 1651, 1620, 1583, 1535, 1349, 1215, 1120. ^1^H NMR (300 MHz, DMSO-*d*_6_) δ 12.39 (s, 1H), 8.63 (s, 1H), 8.39–8.13 (m, 4H), 8.11–7.97 (m, 3H), 7.92 (d, *J* = 8.5 Hz, 1H), 7.32 (d, *J* = 8.5 Hz, 2H), 7.14 (d, *J* = 7.8 Hz, 2H), 3.93 (s, 3H). ^13^C NMR (75 MHz, DMSO) δ 163.77, 162.33, 152.43, 150.89, 146.99, 145.24, 140.37, 138.34, 130.29, 128.58, 125.97, 125.43, 125.04, 124.62, 115.71, 115.65, 114.39, 56.26. Anal.Calcd for C_22_H_17_N_5_O_4_: C 63.61, H 4.13, N 16.68; Found: C 63.52, H 4.26, N 16.72.

### (*E*)-2-(4-methoxyphenyl)-*N'*-((6-nitrobenzo[d][1,3]dioxol-5-yl)methylene)-1H-benzo[d]imidazole-5-carbohydrazide (8o)

Yield 82% (376 mg), light − brown solid: m.p. > 250 °C. IR (KBr) υ: 3251, 1666, 1624, 1596, 1552, 1352, 1261, 1090. ^1^H NMR (300 MHz, DMSO-*d*_6_) δ 12.24 (s, 1H), 8.84 (s, 1H), 8.42 (s, 1H), 8.20 (s, 1H), 8.16 (d, *J* = 8.5 Hz, 2H), 8.00 (d, *J* = 8.7 Hz, 1H), 7.82 (d, *J* = 8.5 Hz, 1H), 7.61 (s, 1H), 7.37 (s, 1H), 7.15 (d, *J* = 7.8 Hz, 2H), 6.27 (s, 2H), 3.87 (s, 3H). ^13^C NMR (75 MHz, DMSO) δ 163.62, 162.86, 152.14, 151.59, 149.28, 145.21, 143.56, 143.39, 138.81, 135.48, 133.10, 130.35, 128.77, 126.22, 126.00, 116.03, 115.54, 114.08, 105.52, 104.35, 56.21. Anal.Calcd for C_23_H_17_N_5_O_6_: C 60.13, H 3.73, N 15.24; Found: C 60.29, H 3.51, N 15.48.

### (*E*)-2-(4-methoxyphenyl)-*N'*-(thiophen-2-ylmethylene)-1H-benzo[d]imidazole-5-carbohydrazide (8p)

Yield 68% (255 mg), white solid: m.p. > 250 °C. IR (KBr) υ: 3248, 1670, 1619, 1585, 1452, 1361, 1195. ^1^H NMR (300 MHz, DMSO-*d*_6_) δ 12.08 (s, 1H), 8.73 (s, 1H), 8.41 (s, 1H), 8.24 (d, *J* = 1.4 Hz, 1H), 8.20 (d, *J* = 9.0 Hz, 2H), 8.09–8.00 (m, 1H), 7.88 (d, *J* = 8.6 Hz, 1H), 7.68 (d, *J* = 5.1 Hz, 1H), 7.45 (dd, *J* = 3.8, 1.2 Hz, 1H), 7.32 (td, *J* = 5.1, 1.2 Hz, 1H), 7.24 (d, *J* = 9.0 Hz, 2H), 3.88 (s, 3H). ^13^C NMR (75 MHz, DMSO) δ 164.05, 162.64, 151.17, 144.98, 143.96, 139.44, 138.96, 134.12, 131.85, 131.62, 131.18, 130.72, 128.82, 128.39, 125.99, 115.74, 114.91, 113.93, 56.31. Anal.Calcd for C_20_H_16_N_4_O_2_S: C 63.81, H 4.28, N 14.88, S 8.52; Found: C 63.69, H 4.52, N 14.53, S 8.41.

### α-Glucosidase inhibition assay

The α-glucosidase inhibitory effects of benzimidazole-Schiff base derivatives **8a–p** were evaluated under the basis of our previously reported method^29^. According this protocol, 20 μL of enzyme solution (α-glucosidase from *Saccharomyces cerevisiae*, EC3.2.1.20, 20 U/mg), 20 μL of test compounds **8a–p** with various concentrations, and 135 μL of potassium phosphate buffer were added and incubated in the 96-well plate for 10 min at 37 °C. Later on, 25 μL of substrate (*p*-nitrophenyl glucopyranoside, 4 mM) was added to each well of the plate and incubation was continued for 20 min at 37 °C. Next, absorbance was measured at 405 nm by spectrophotometer (Gen5, Power wave xs2, BioTek, USA), and IC_50_ value for each tested compound was calculated by taking advantage of the nonlinear regression curve (logit method).

### Enzyme kinetic studies

The mode of inhibition of the most active compound **8p**, identified with the lowest IC_50_, was investigated against α-glucosidase in different concentrations (0, 20, 40 and 60 µM) of *p*-nitrophenyl glucopyranoside (2–10 mM) as substrate. A Lineweaver–Burk plot was generated to identify the type of inhibition and the Michaelis–Menten constant (*K*_m_) value was determined from plot between reciprocal of the substrate concentration (1/[S]) and reciprocal of enzyme rate (1/V) over various inhibitor concentrations. Experimental inhibitor constant (*K*_i_) value was constructed by secondary plots of the inhibitor concentration [I] versus *K*_m_.

### Docking study

Maestro Molecular Modeling platform (version 12.8) by Schrödinger, LLC was performed to uncover out the interaction mode of the best active structures over α-glycosidase enzyme^20,21^. The protein 3D structure was implemented according to our previous study as a result of homology modeled based on high structural identity and sequence similarity with α-glucosidase (α-1,4-glucosidase) from *S. cerevisiae* (PDB code 3A4A).

The 2D representation of the synthesized compounds were drawn in Marvin 15.10.12.0 program (http://www.chemaxon.com) and converted into pdb file. The Protein Preparation Wizard and the LigPrep module were used to prepare protein and ligand structure properly. The missing side chains of the proteins were filled using the Prime tool and missing residues were updated.

The accurate side-chain and backbone flexibility during ligand binding at the active site of α-glycosidase enzyme were predicted by IFD method using Glide software (Schrödinger LLC 2018, USA). As the kinetic study revealed competitive type inhibition mechanism against enzyme, the α-glucosidase active site was used to generate the grid for IFD calculation. The maximum 20 poses with receptor and ligand van der waals radii of 0.7 and 0.5, respectively considered. Residues within 5 Å of the α-d-glucose at the active site were refined followed by side-chain optimization. Structures whose Prime energy is more than 30 kcal/mol are eliminated based on extra precious Glide docking.

### MD simulation

MD simulation was performed by using the Desmond v5.3 module implemented in Maestro interface (from Schrödinger 2018‐4 suite). The appropriate pose for MD simulation procedure of the compounds was achieved by IFD method.

In order to build the system for MD simulation, the protein–ligand complexes were solvated with SPC explicit water molecules and placed in the center of an orthorhombic box of appropriate size in the Periodic Boundary Condition. Sufficient counter‐ions and a 0.15 M solution of NaCl were also utilized to neutralize the system and to simulate the real cellular ionic concentrations, respectively. The MD protocol involved minimization, pre-production, and finally production MD simulation steps. In the minimization procedure, the entire system was allowed to relax for 2500 steps by the steepest descent approach. Then the temperature of the system was raised from 0 to 310 K with a small force constant on the enzyme in order to restrict any drastic changes. MD simulations were performed via NPT (constant number of atoms, constant pressure i.e. 1.01325 bar and constant temperature i.e. 310 K) ensemble. The Nose‐Hoover chain method was used as the default thermostat with 1.0 ps interval and Martyna–Tobias–Klein as the default barostat with 2.0 ps interval by applying isotropic coupling style. Long‐range electrostatic forces were calculated based on Particle‐mesh‐based Ewald approach with the he cut‐off radius for columbic forces set to 9.0 Å. Finally, the system subjected to produce MD simulations for 30 ns for protein–ligand complex. During the simulation every 1000 ps of the actual frame was stored. The dynamic behavior and structural changes of the systems were analyzed by the calculation of the root mean square deviation (RMSD). Subsequently, the energy-minimized structure calculated from the equilibrated trajectory system was evaluated for investigation of each ligand–protein complex interaction.

### In silico druglikeness, ADME, and toxicity studies

In silico druglikeness/ADME/Tox studies of the most potent compounds were performed using by preADMET online server (http://preadmet.bmdrc.org/)^27^.

### In vitro cytotoxicity assay

In vitro cytotoxicity of the most potent compounds was evaluated by MTT assay in triplicate according to the literature^28^.

## Supplementary Information

Supplementary Information.

## Data Availability

The datasets used or analysed during the current study are available from the corresponding author on reasonable request.
