# Supplementary material for: Docking study, molecular dynamic, synthesis, anti-α-glucosidase assessment, and ADMET prediction of new benzimidazole-Schiff base derivatives
Source: Sci Rep. 2022 Sep 1;12:14870. doi: 10.1038/s41598-022-18896-0 (PMC9437094; doi:10.1038/s41598-022-18896-0)

**Support information**

**Docking study, molecular dynamic, synthesis, anti-α-glucosidase assessment, and ADMET prediction of new benzimidazole-Schiff base derivatives**

Homa azizian^1^, Keyvan Pedrood^2^, Ali Moazzam^2^, Yousef Valizadeh^2^, Kimia Khavaninzadeh^2^, Ali Zamani^3^, Maryam Mohammadi-Khanaposhtani^4^*, Somayeh Mojtabavi^5^, Mohammad Ali Faramarzi^5^, Samanesadat Hosseini^6^, Yaghoub Sarrafi^3^, Hossein adibi^2^, Bagher Larijani^2^, Hossein Rastegar^7^, Mohammad Mahdavi^2^*

^1^Department of Medicinal Chemistry, School of Pharmacy, Iran University of Medical Sciences, Tehran, Iran. ^2^Endocrinology and Metabolism Research Center, Endocrinology and Metabolism Clinical Sciences Institute, Tehran University of Medical Sciences, Tehran, Iran. ^✉^e-mail: [momahdavi@tums.ac.ir](mailto:momahdavi@tums.ac.ir) (M. Mahdavi). ^3^Faculty of Chemistry, University of Mazandaran, Babolsar, Iran. ^4^Cellular and Molecular Biology Research Center, Health Research Institute, Babol University of Medical Sciences, Babol, Iran. ^✉^e-mail: [maryammoha@gmail.com](mailto:maryammoha@gmail.com) (M. Mohammadi-Khanaposhtani). ^5^Department of Pharmaceutical Biotechnology, Faculty of Pharmacy, Tehran University of Medical Sciences, Tehran, Iran. ^6^Shahid Beheshti University of Medical Sciences, Tehran, Iran. ^7^Cosmetic products research center, Iranian food and drug administration, MOHE, Tehran, Iran.

(*E*)-*N'*-benzylidene-2-(4-methoxyphenyl)-1*H*-benzo[d]imidazole-5-carbohydrazide (**8a**)


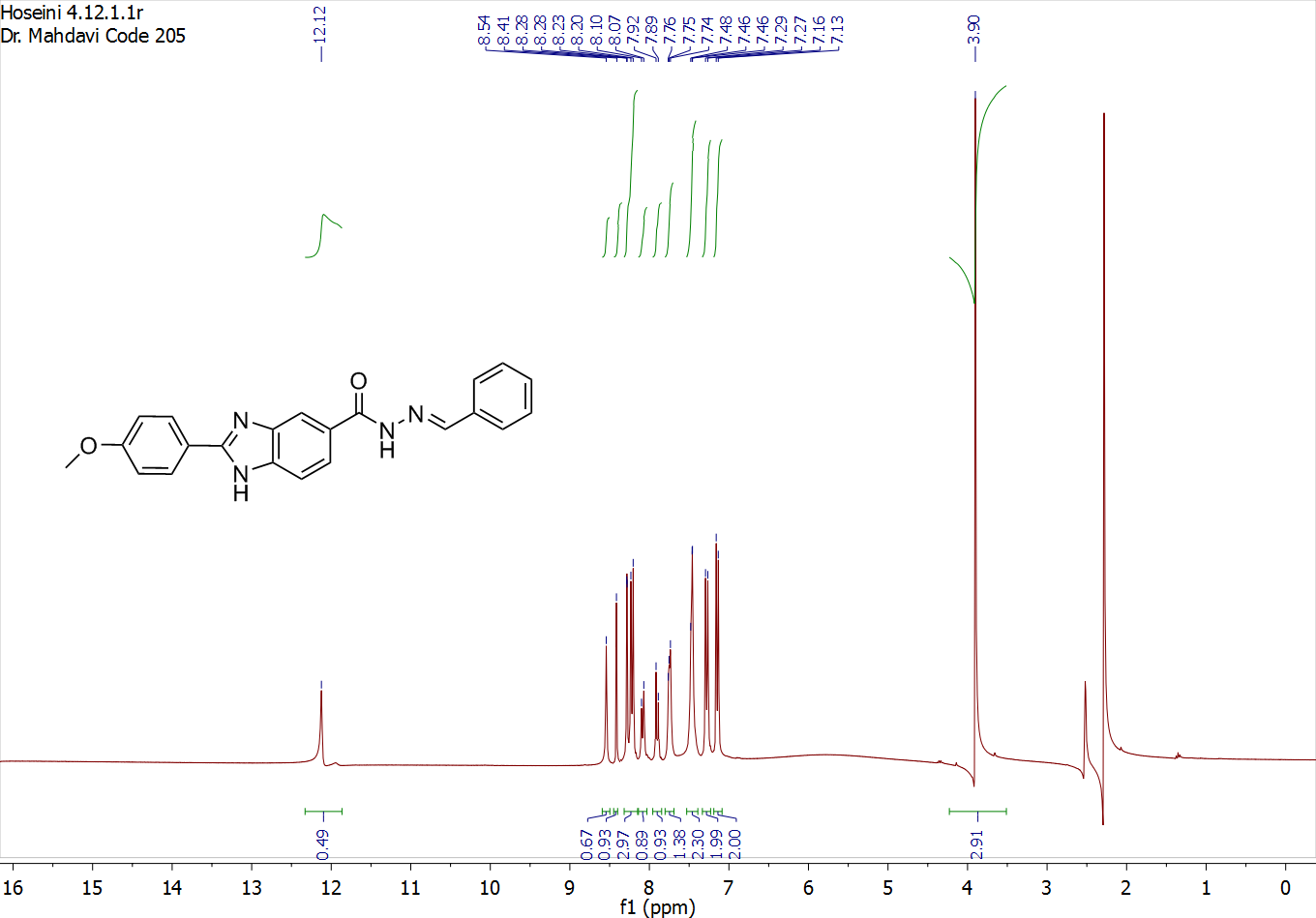


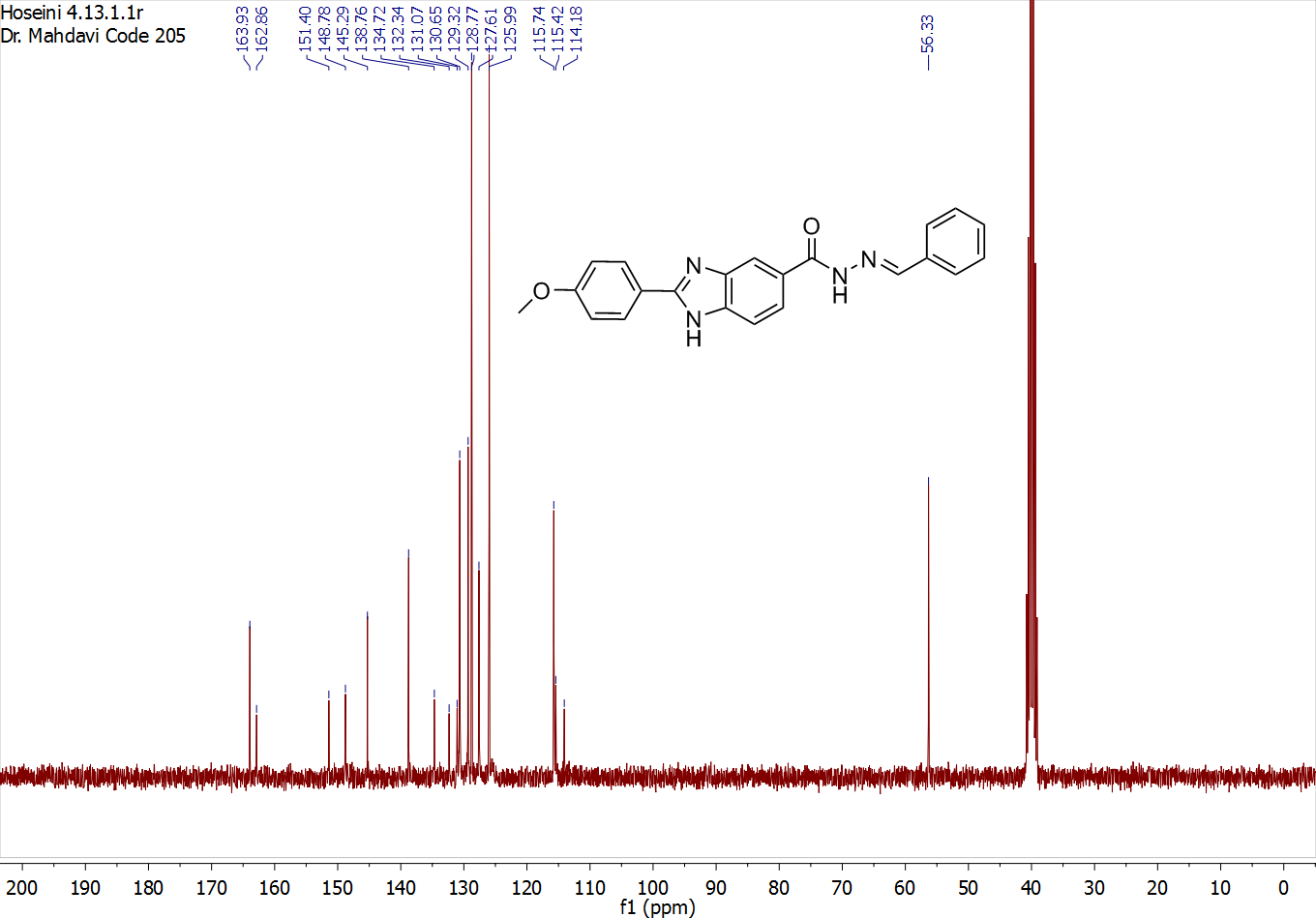


(*E*)-2-(4-methoxyphenyl)-*N'*-(4-methylbenzylidene)-1*H*-benzo[d]imidazole-5-carbohydrazide (**8b**)


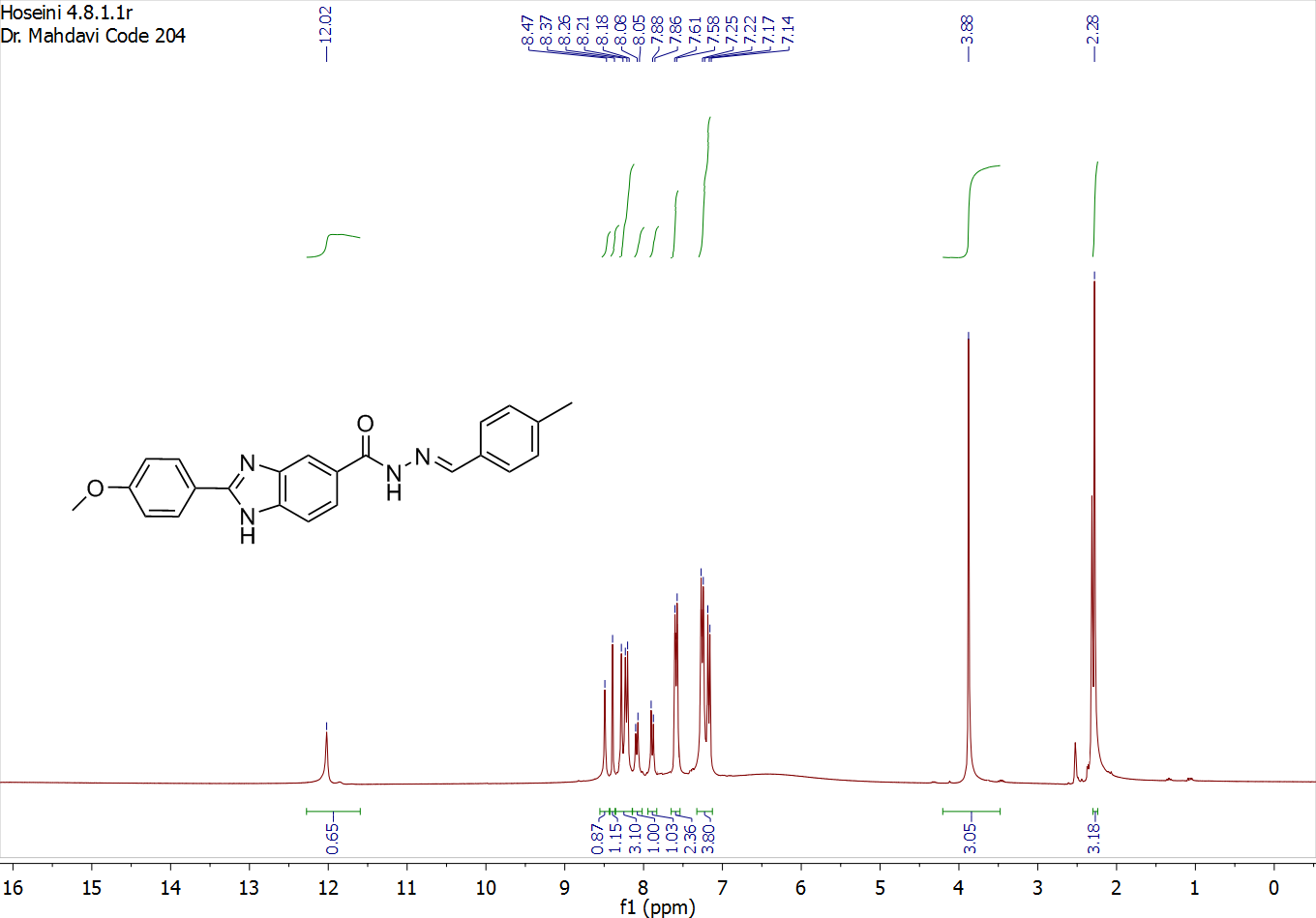


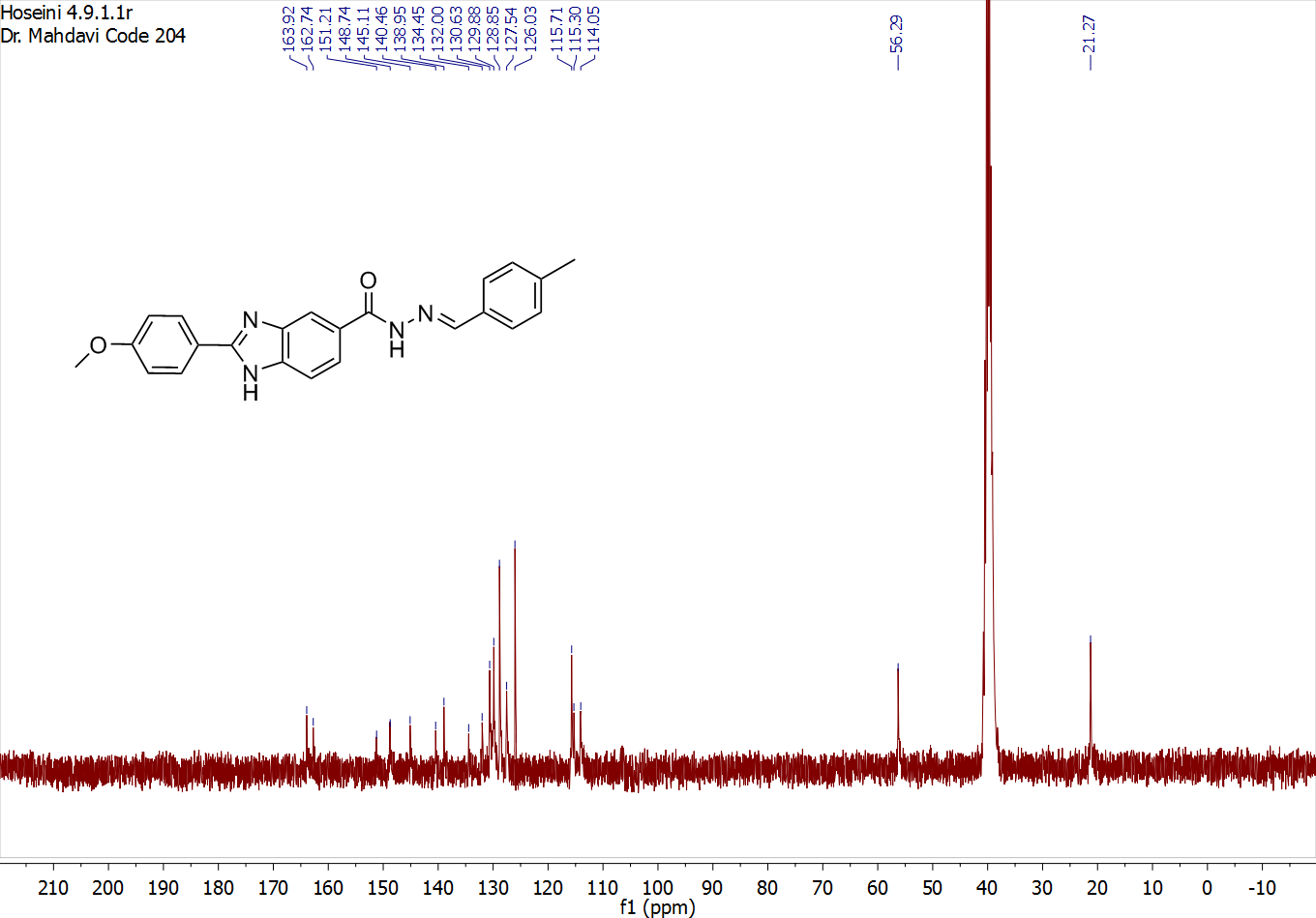


(*E*)-*N'*-(4-methoxybenzylidene)-2-(4-methoxyphenyl)-1*H*-benzo[d]imidazole-5-carbohydrazide (**8c**)
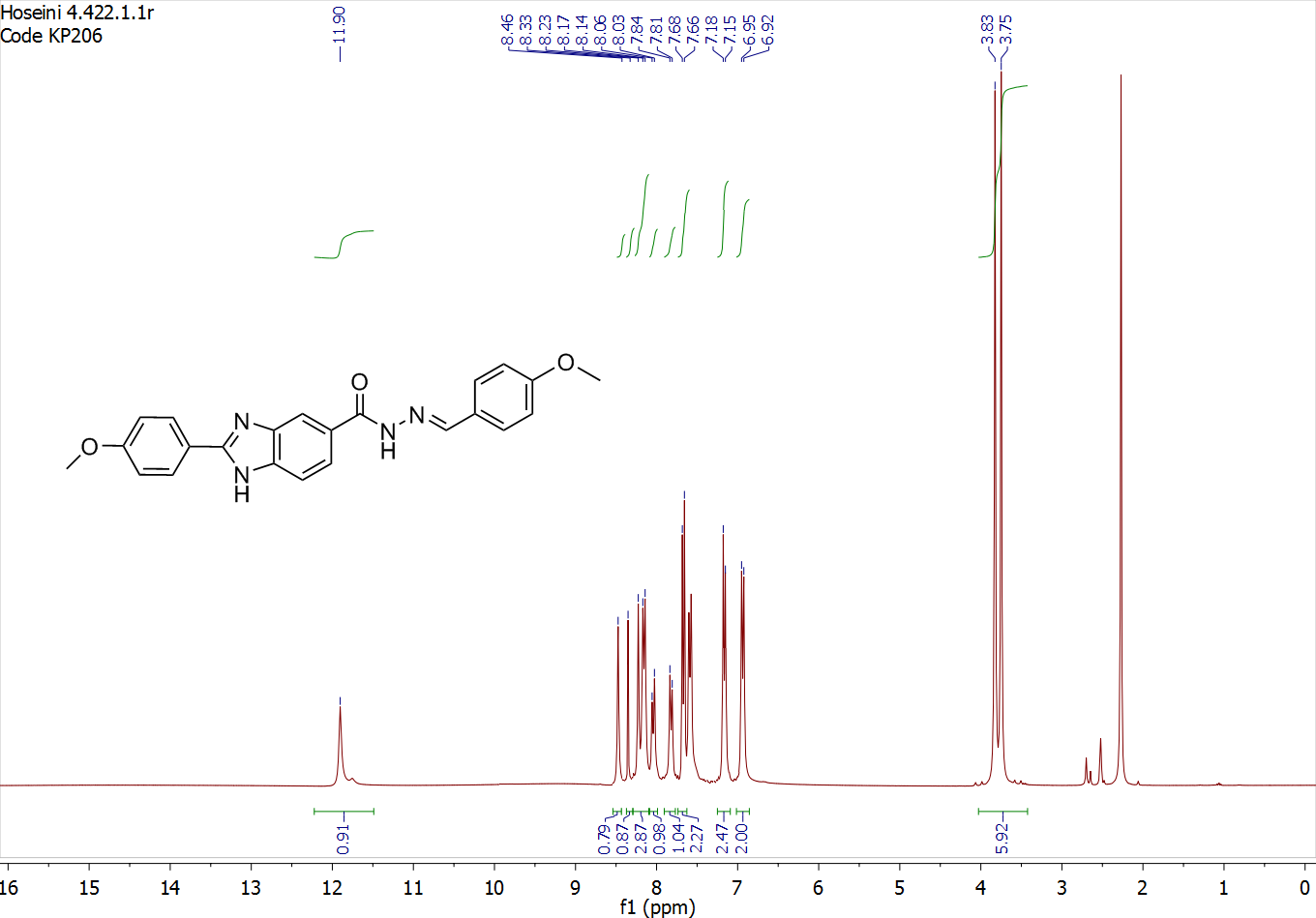
)


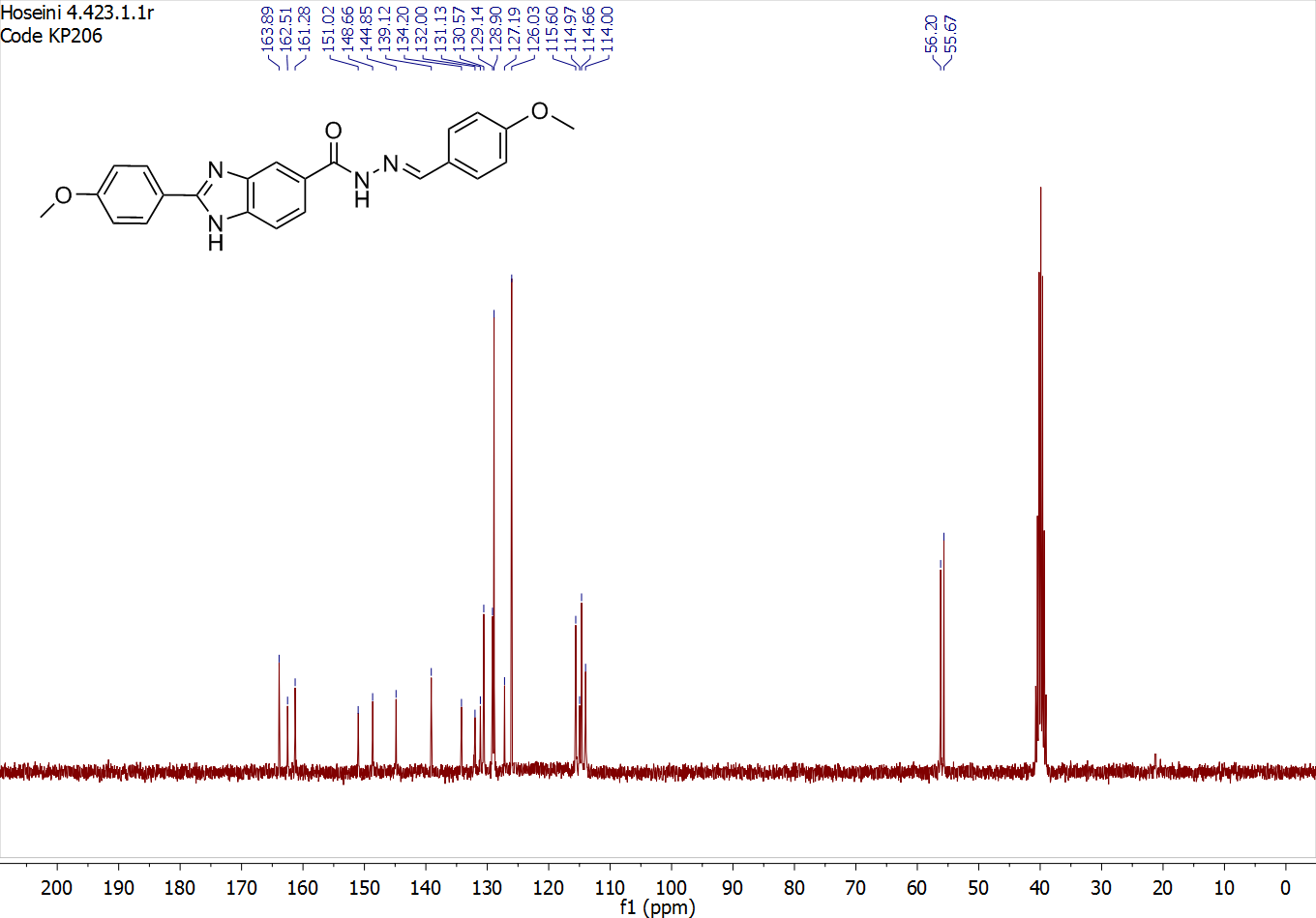


(*E*)-2-(4-methoxyphenyl)-*N'*-(3,4,5-trimethoxybenzylidene)-1*H*-benzo[d]imidazole-5-carbohydrazide (**8d**)


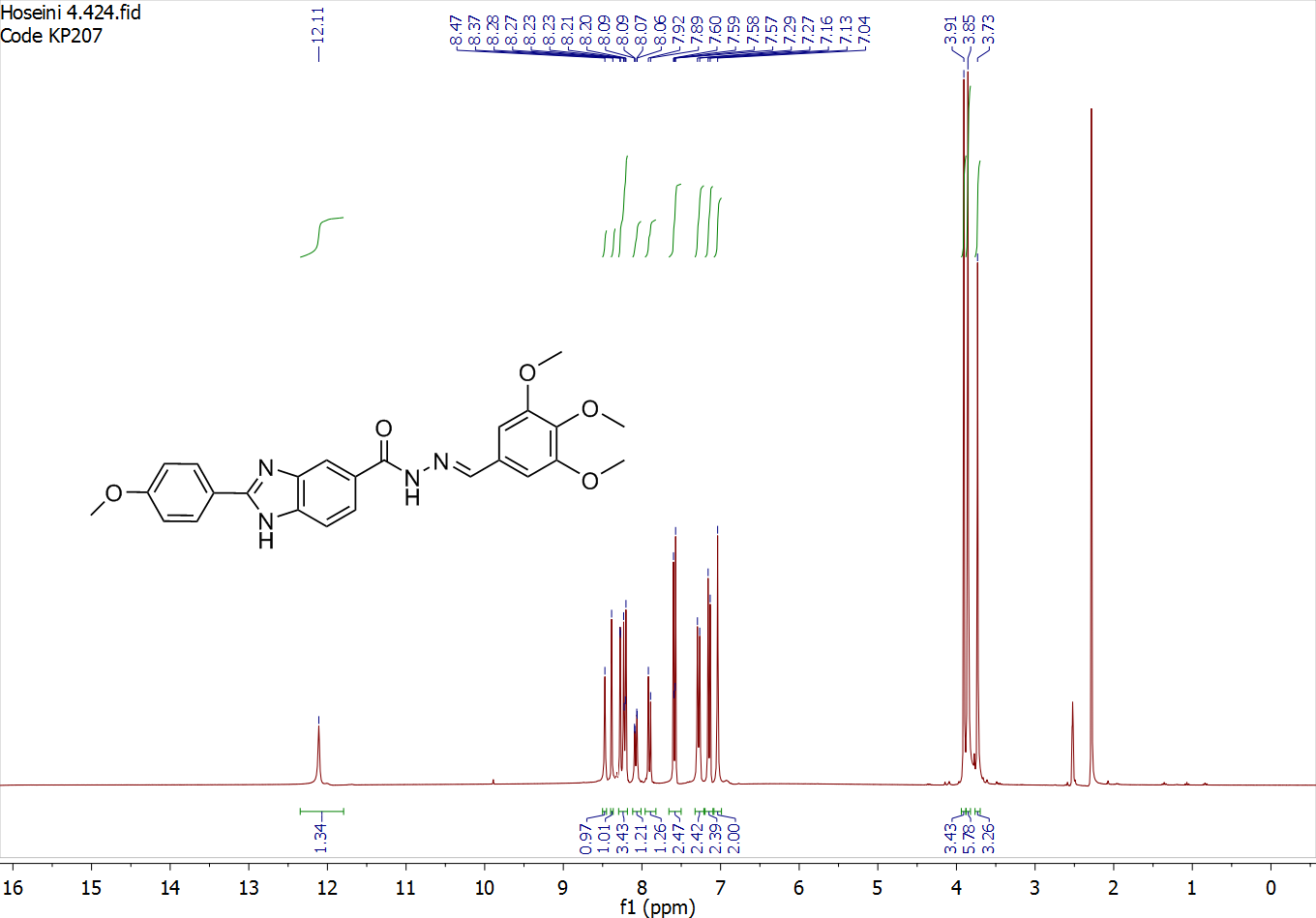


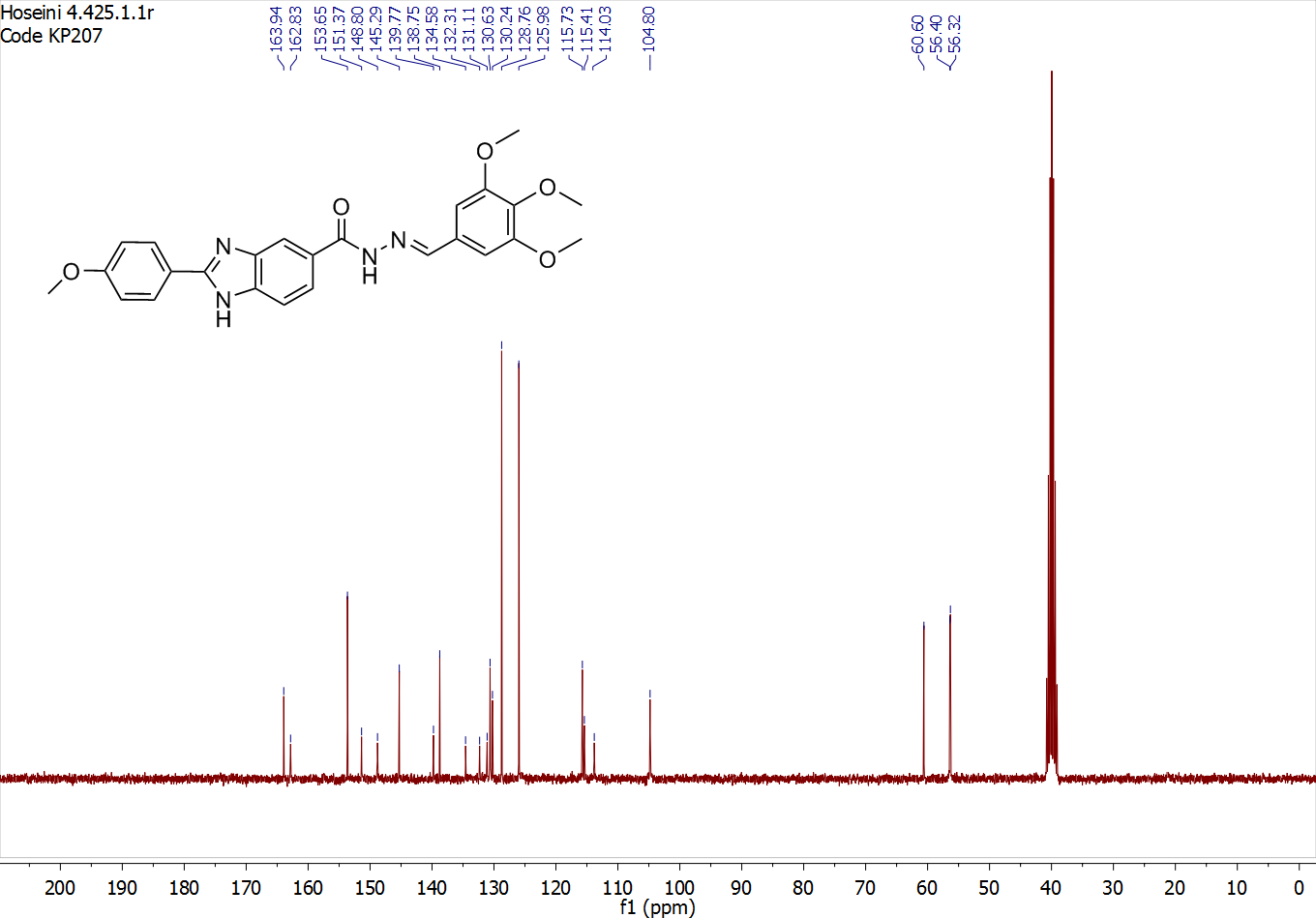


(*E*)-2-(4-methoxyphenyl)-*N'*-(3-phenoxybenzylidene)-1*H*-benzo[d]imidazole-5-carbohydrazide (**8e**)


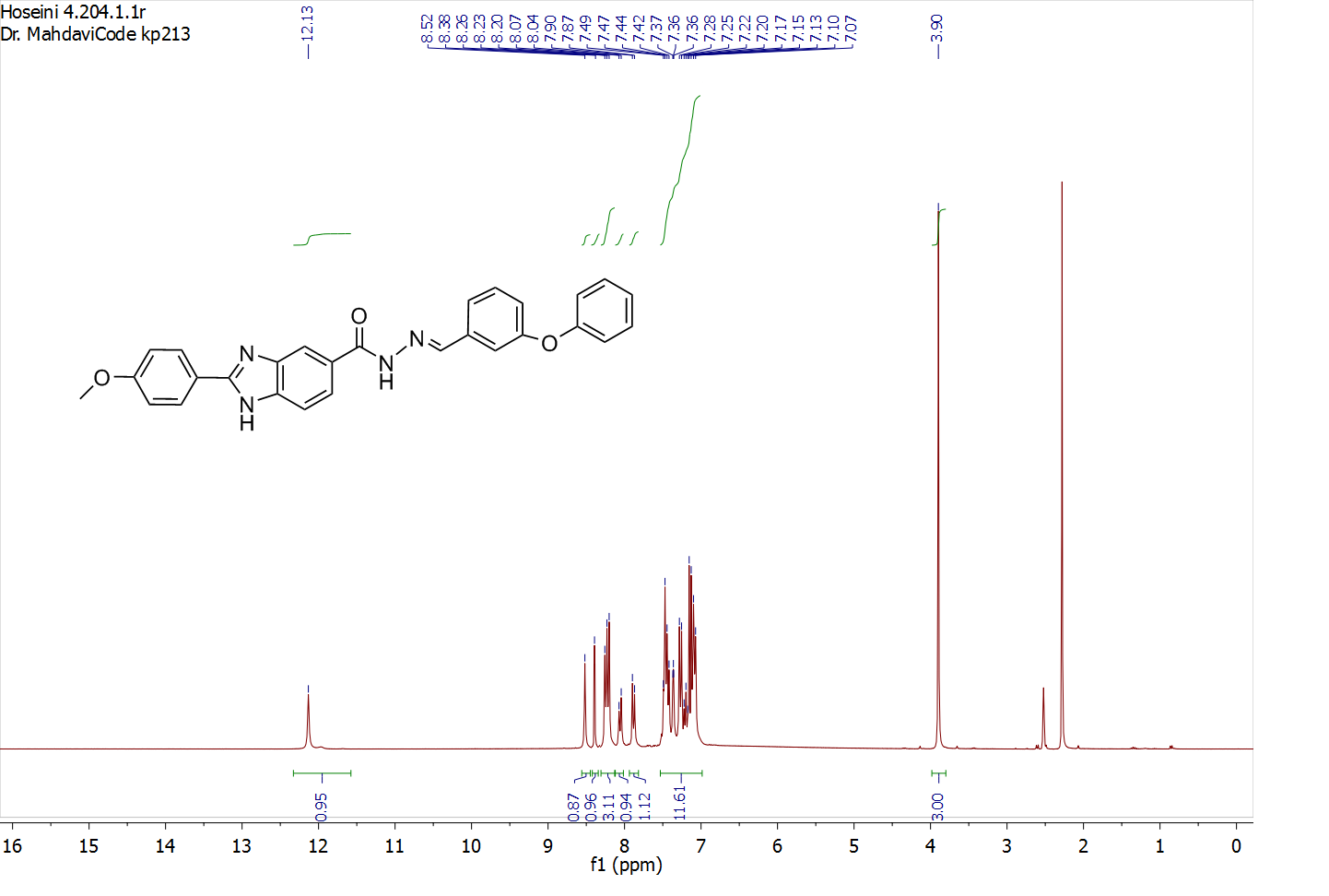


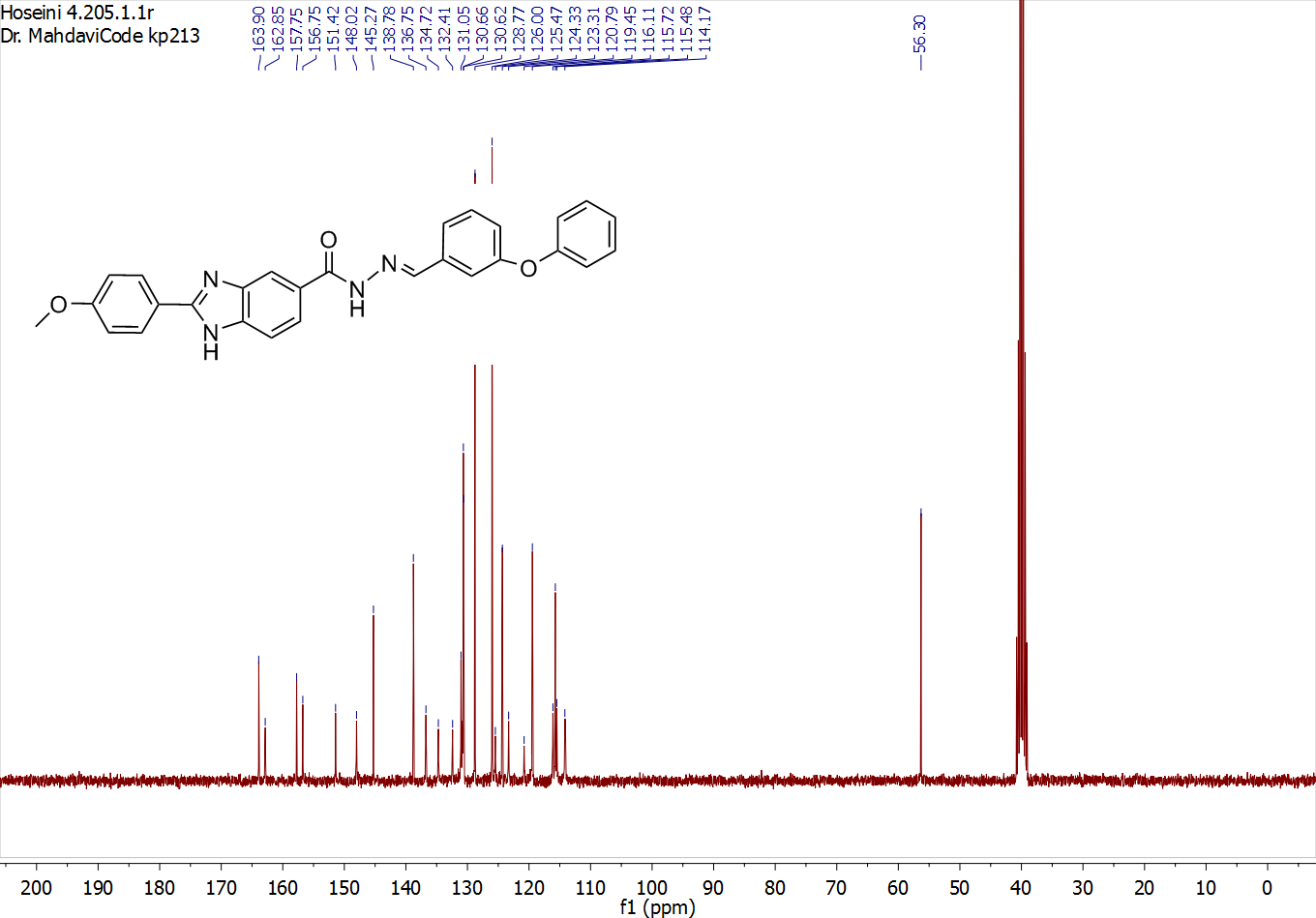


(*E*)-*N'*-(3-hydroxybenzylidene)-2-(4-methoxyphenyl)-1*H*-benzo[d]imidazole-5-carbohydrazide (**8f**)


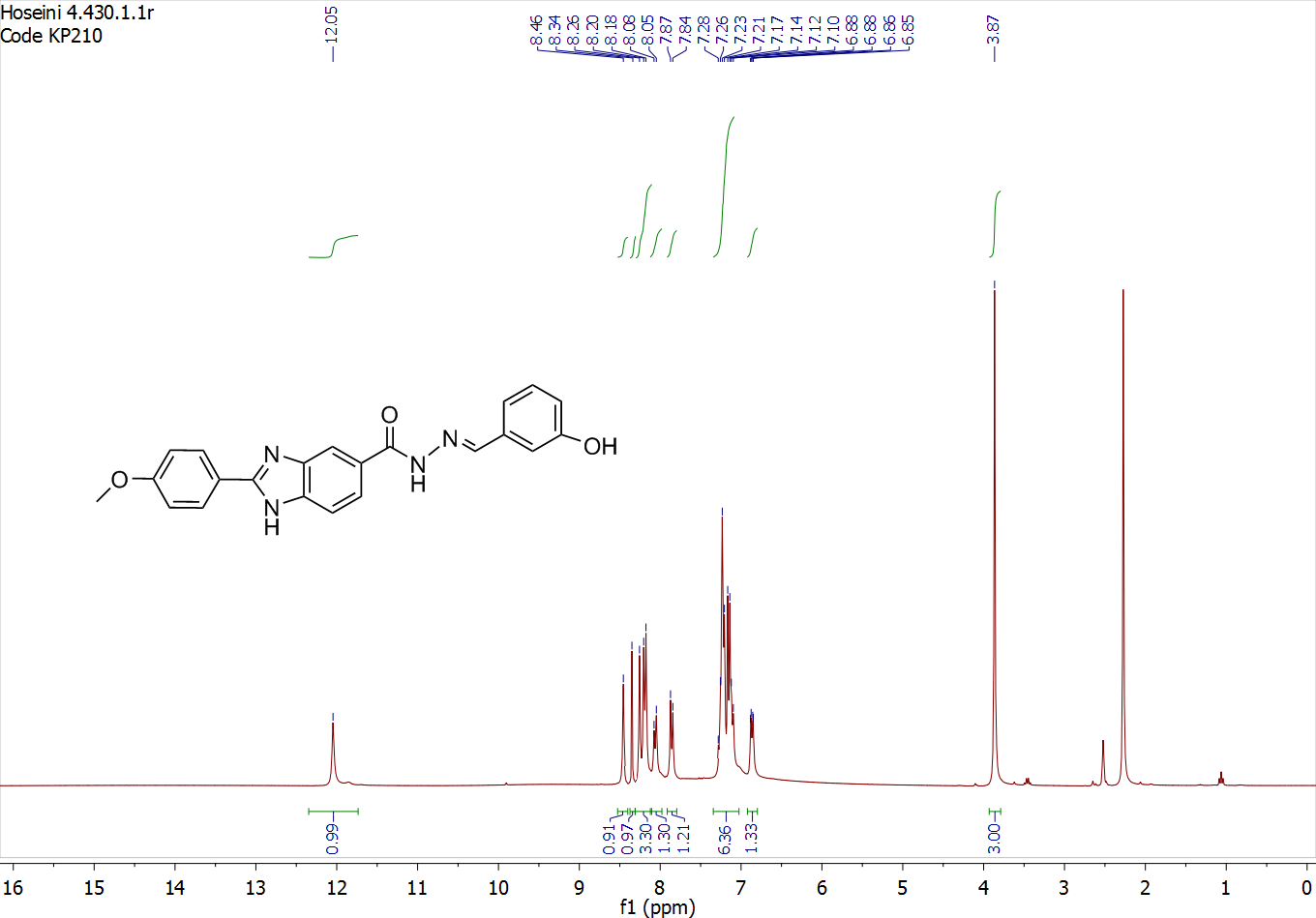


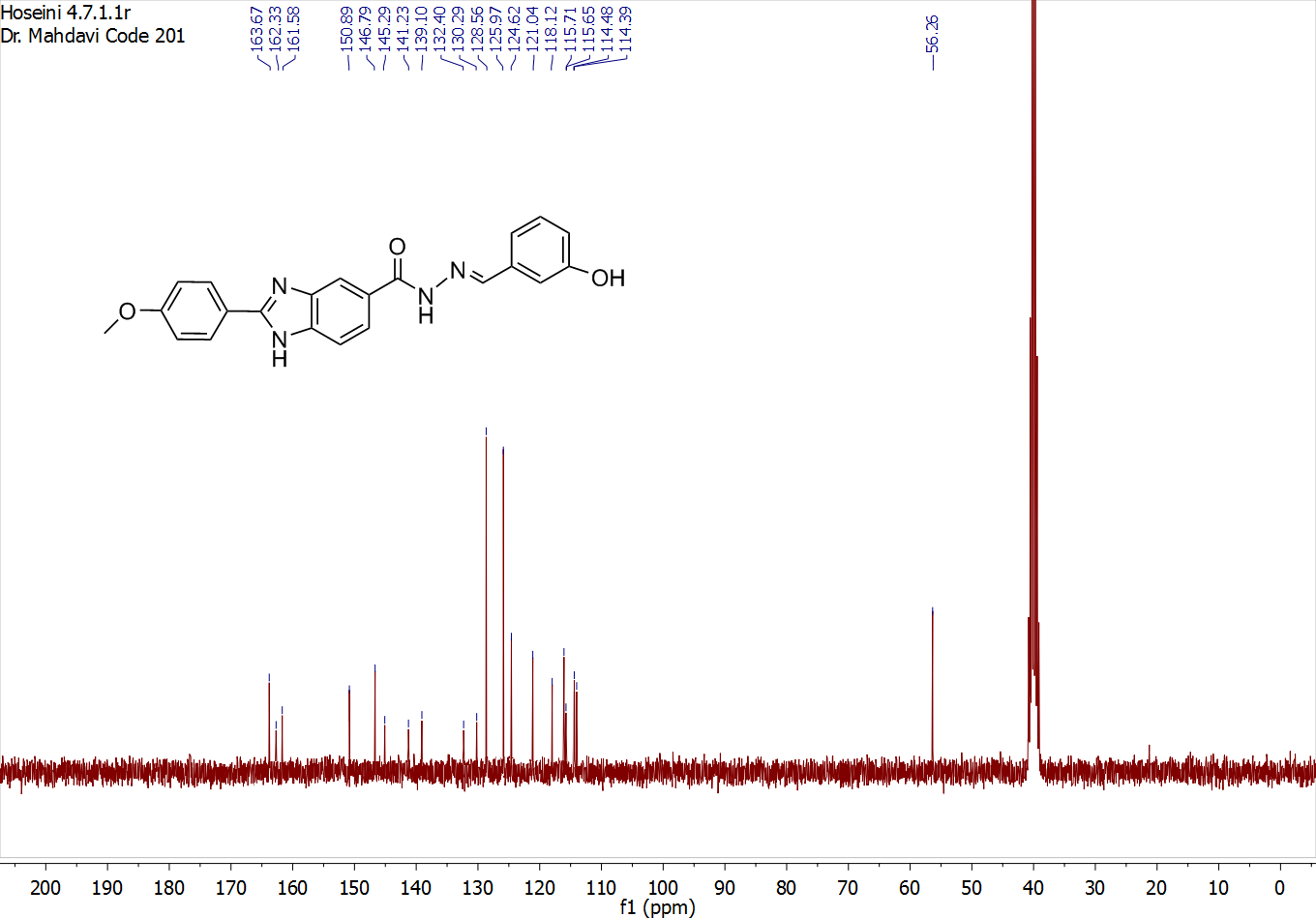


(*E*)-*N'*-(4-hydroxy-3-methoxybenzylidene)-2-(4-methoxyphenyl)-1*H*-benzo[d]imidazole-5-carbohydrazide (**8g**)


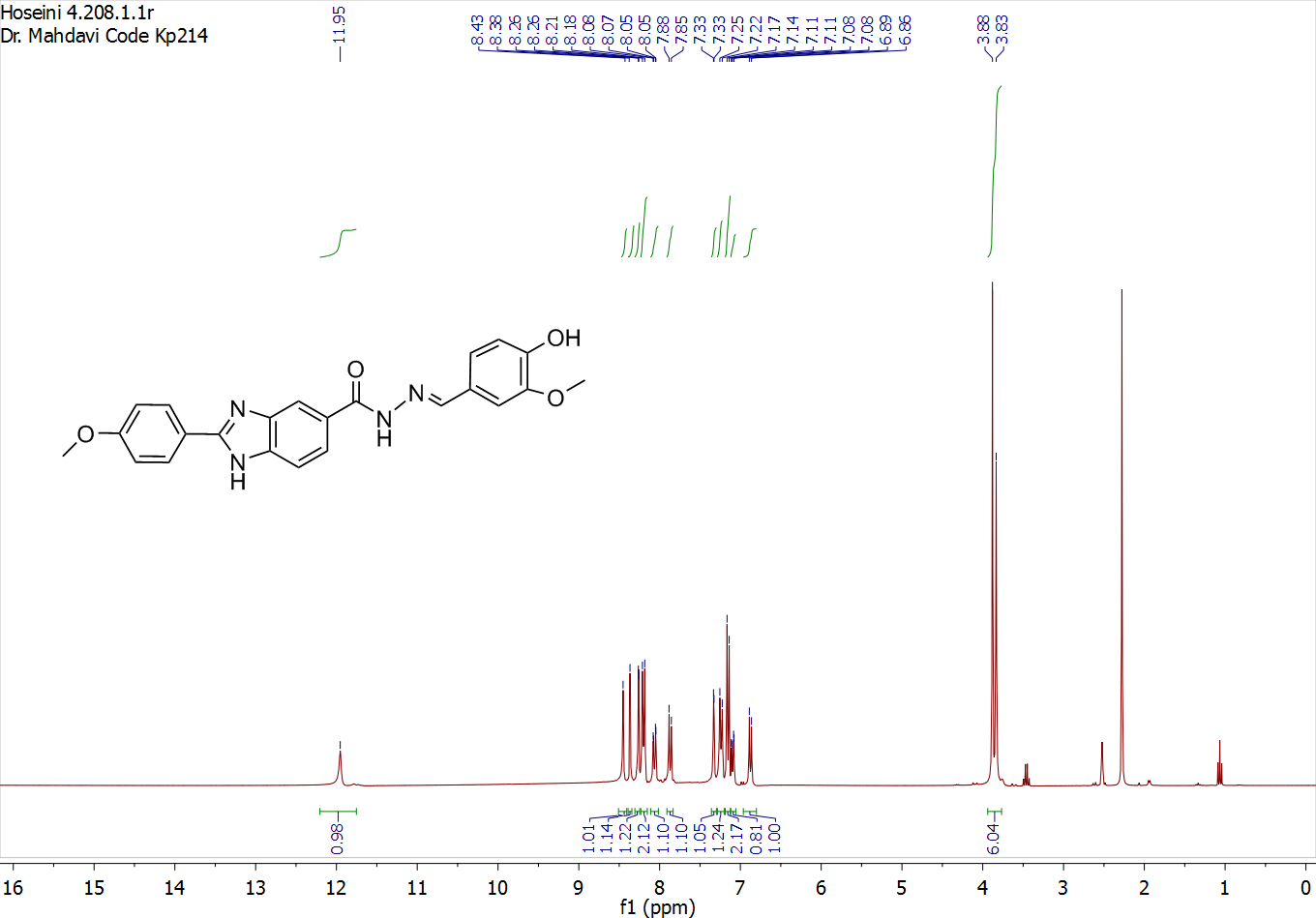


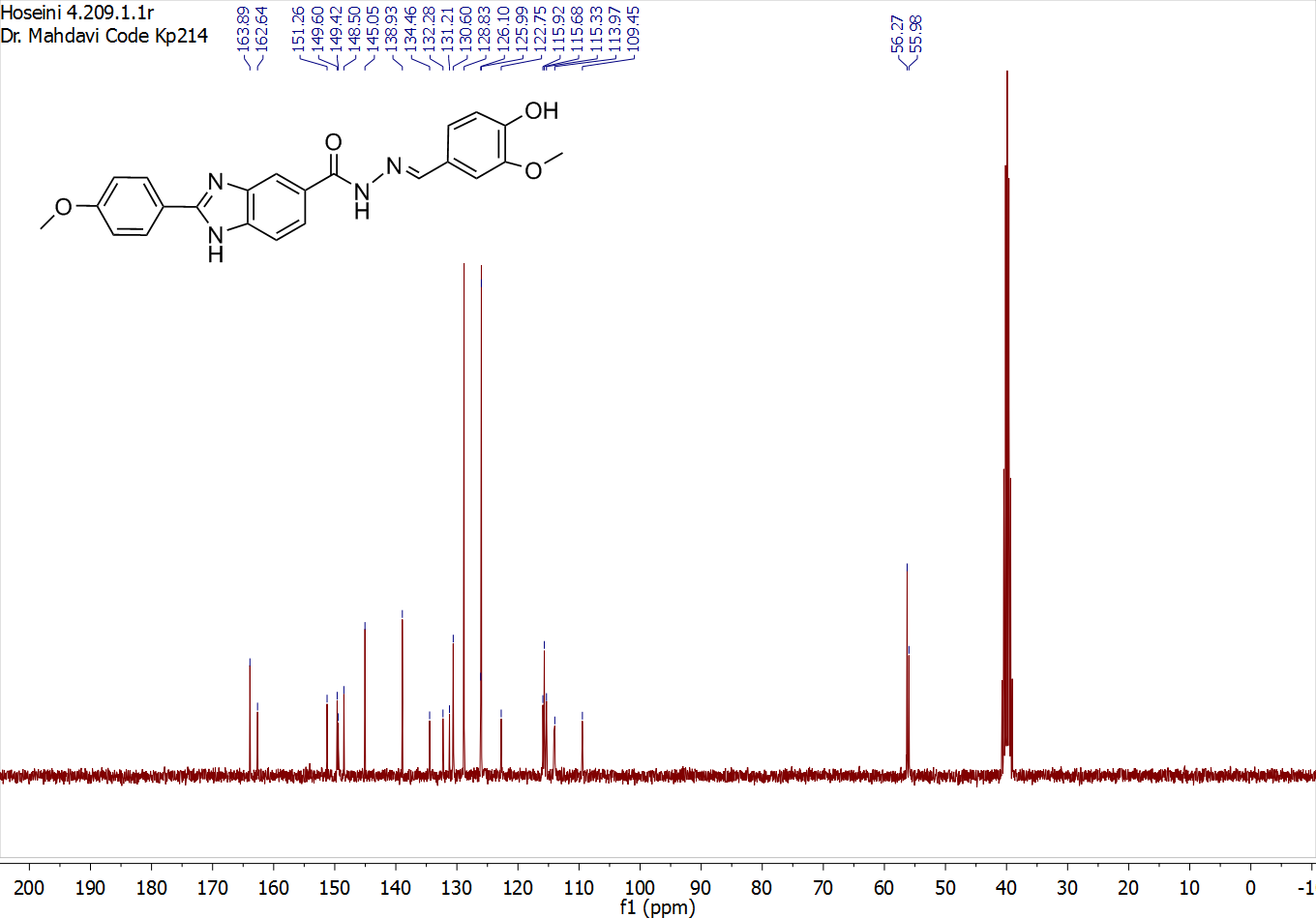


(*E*)-*N'*-(2-fluorobenzylidene)-2-(4-methoxyphenyl)-1*H*-benzo[d]imidazole-5-carbohydrazide (**8h**)


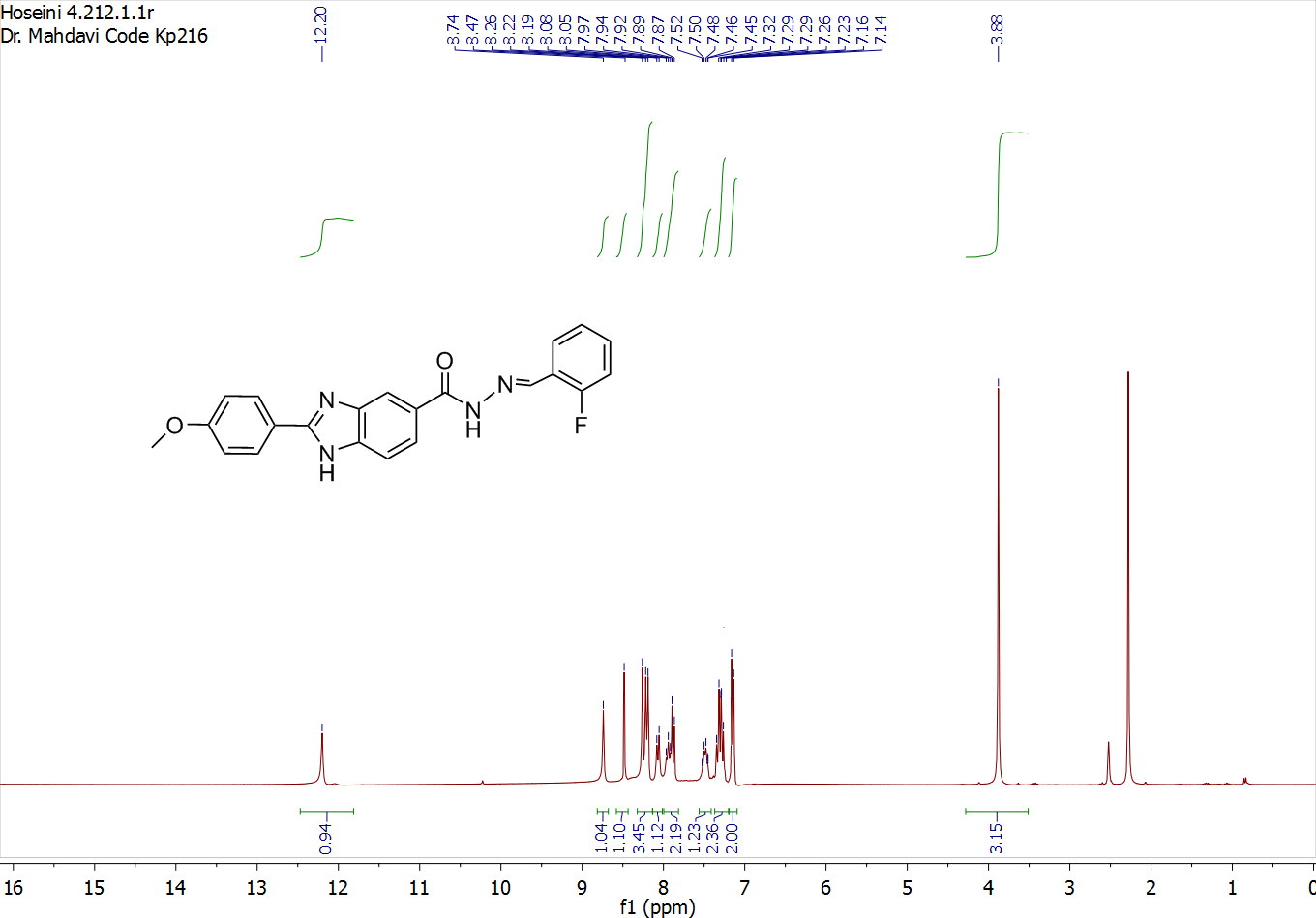


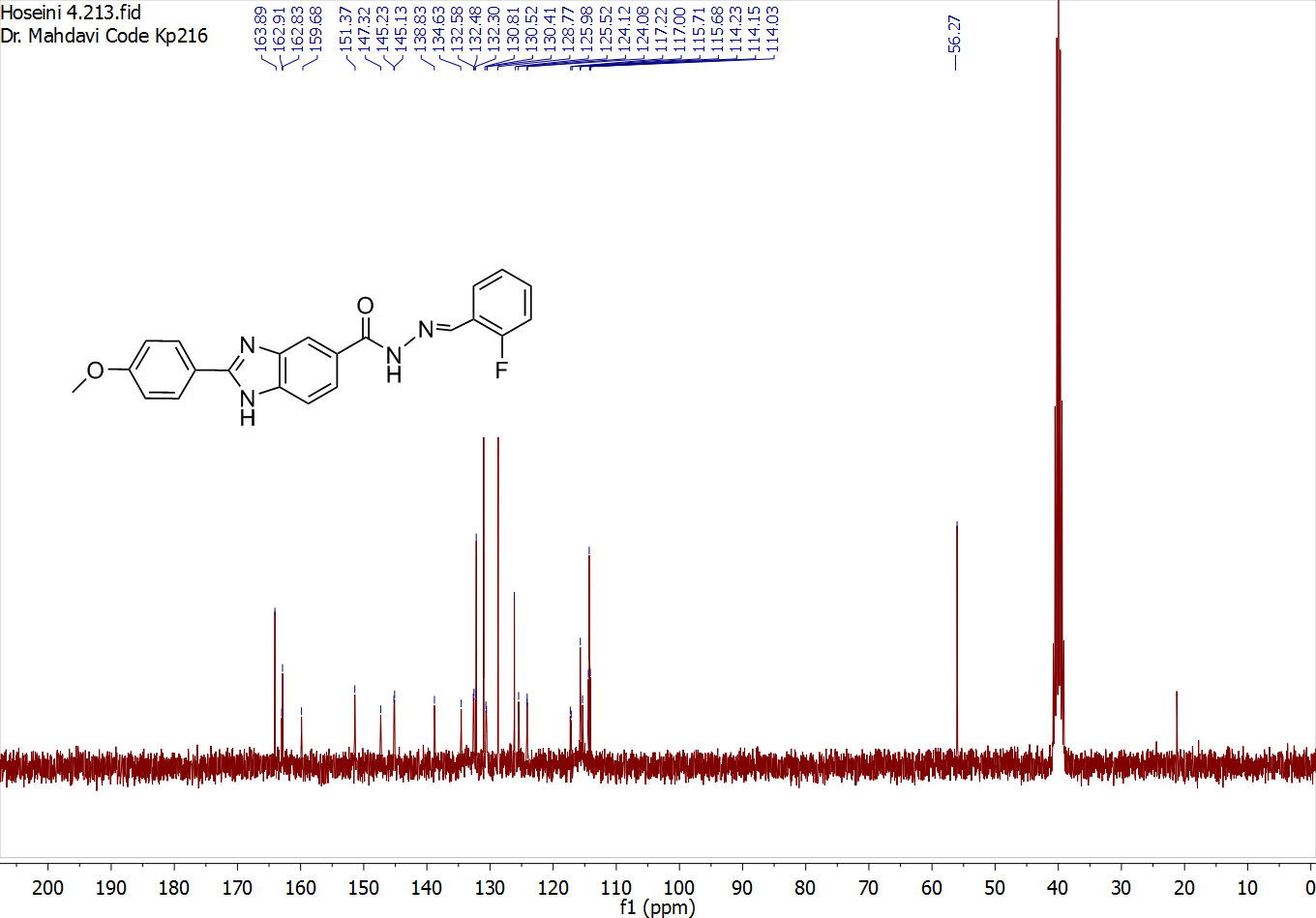


(*E*)-*N'*-(3-fluorobenzylidene)-2-(4-methoxyphenyl)-1*H*-benzo[d]imidazole-5-carbohydrazide (**8i**)


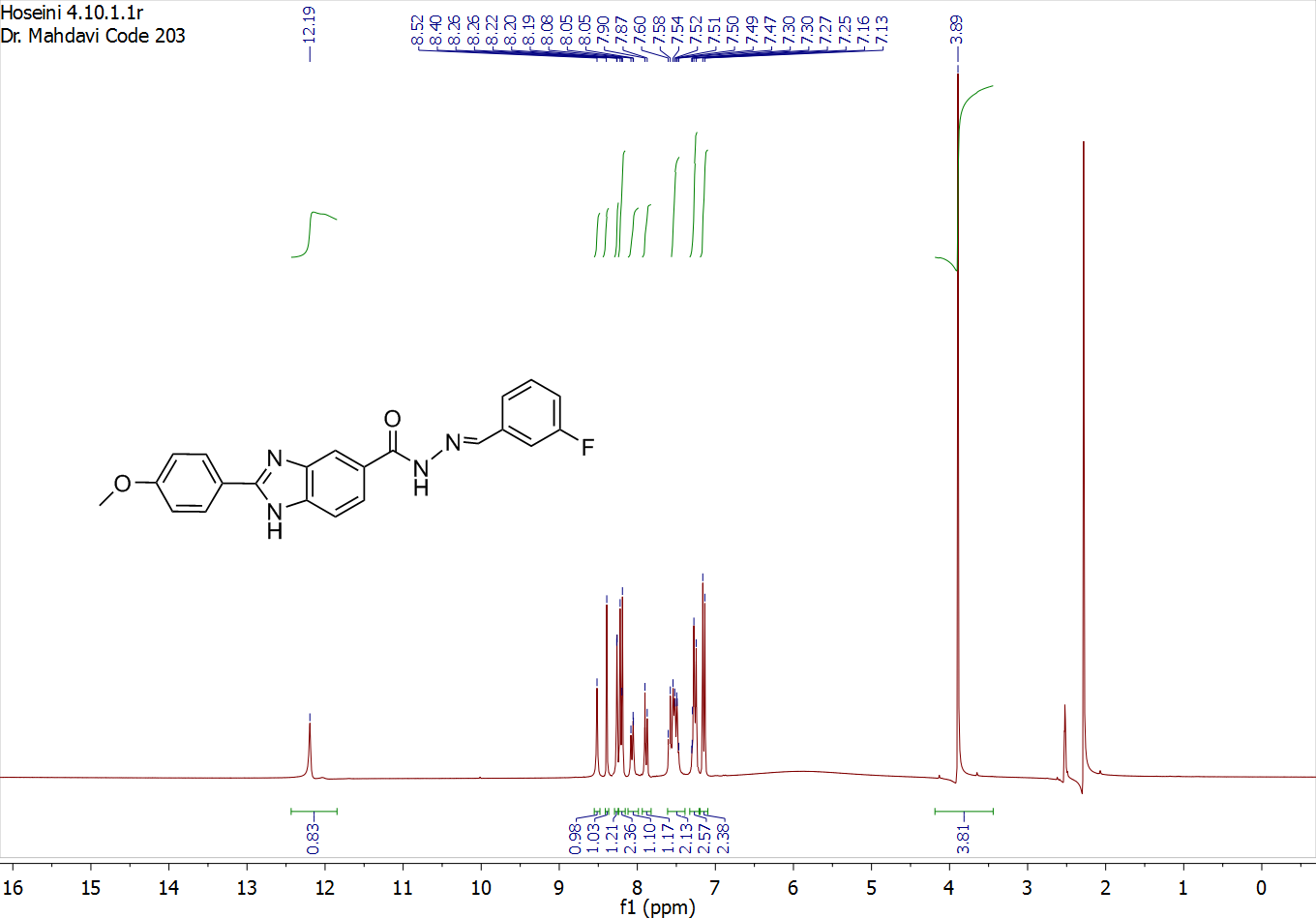


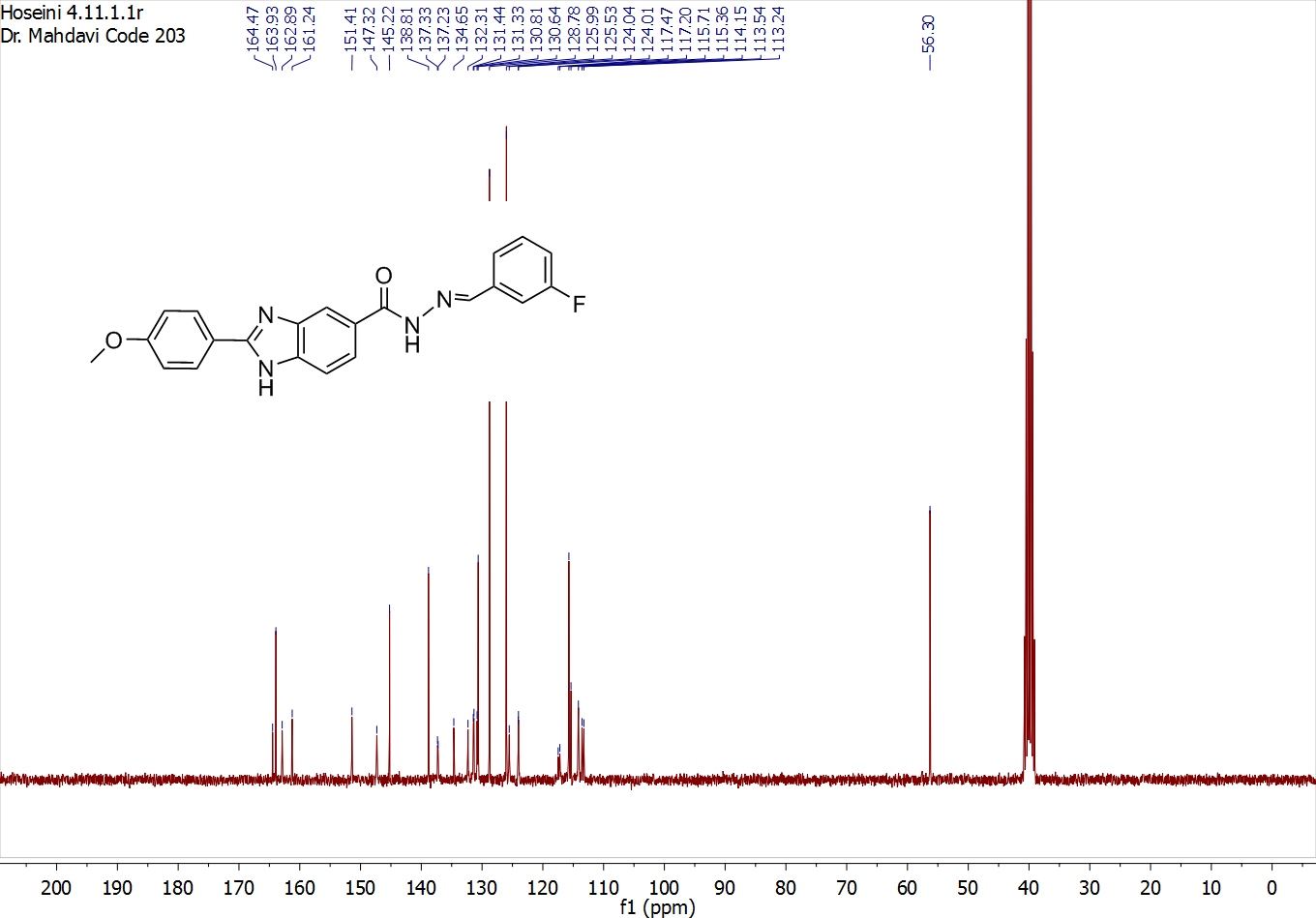


(*E*)-*N'*-(4-fluorobenzylidene)-2-(4-methoxyphenyl)-1*H*-benzo[d]imidazole-5-carbohydrazide (**8j**)


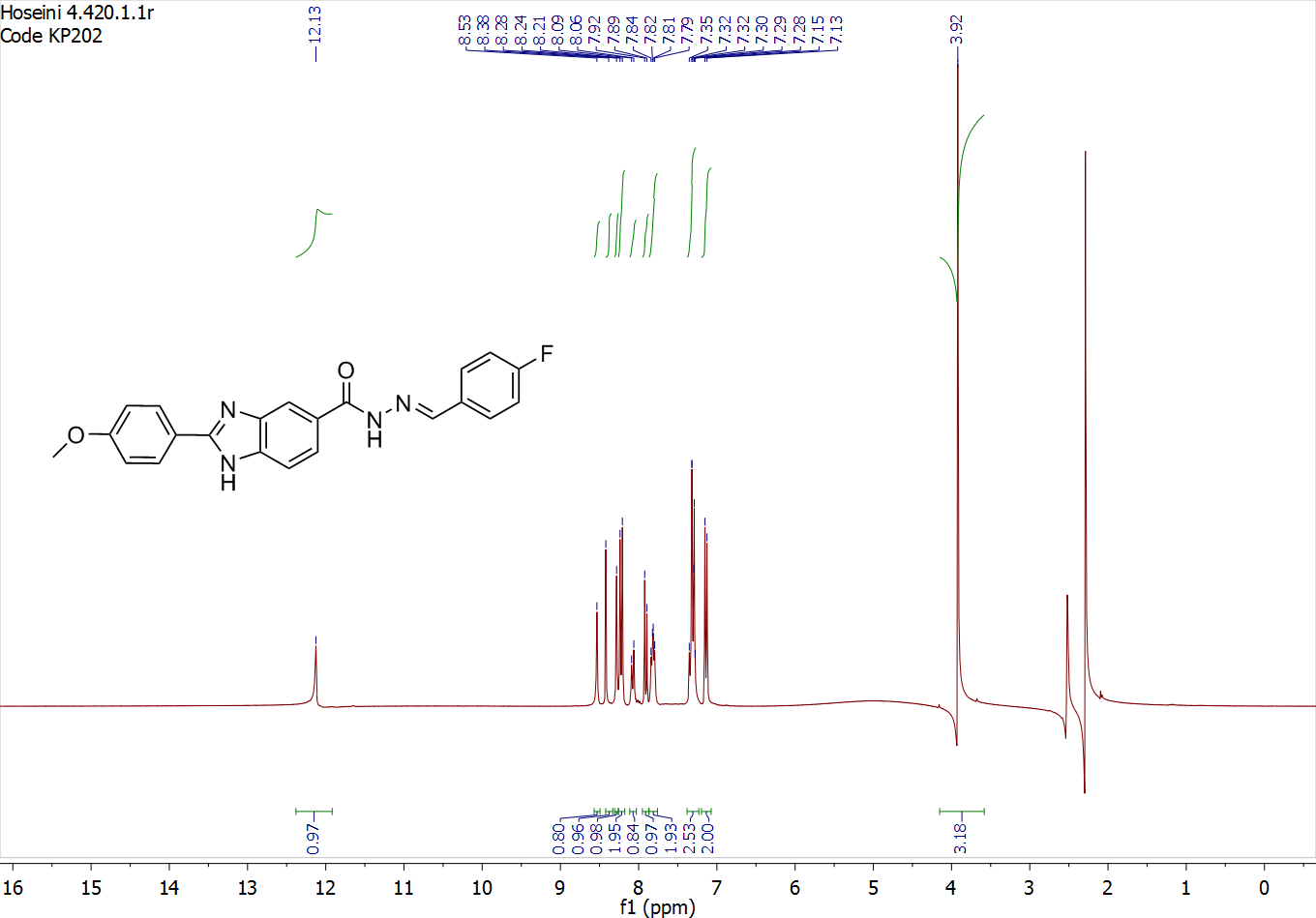


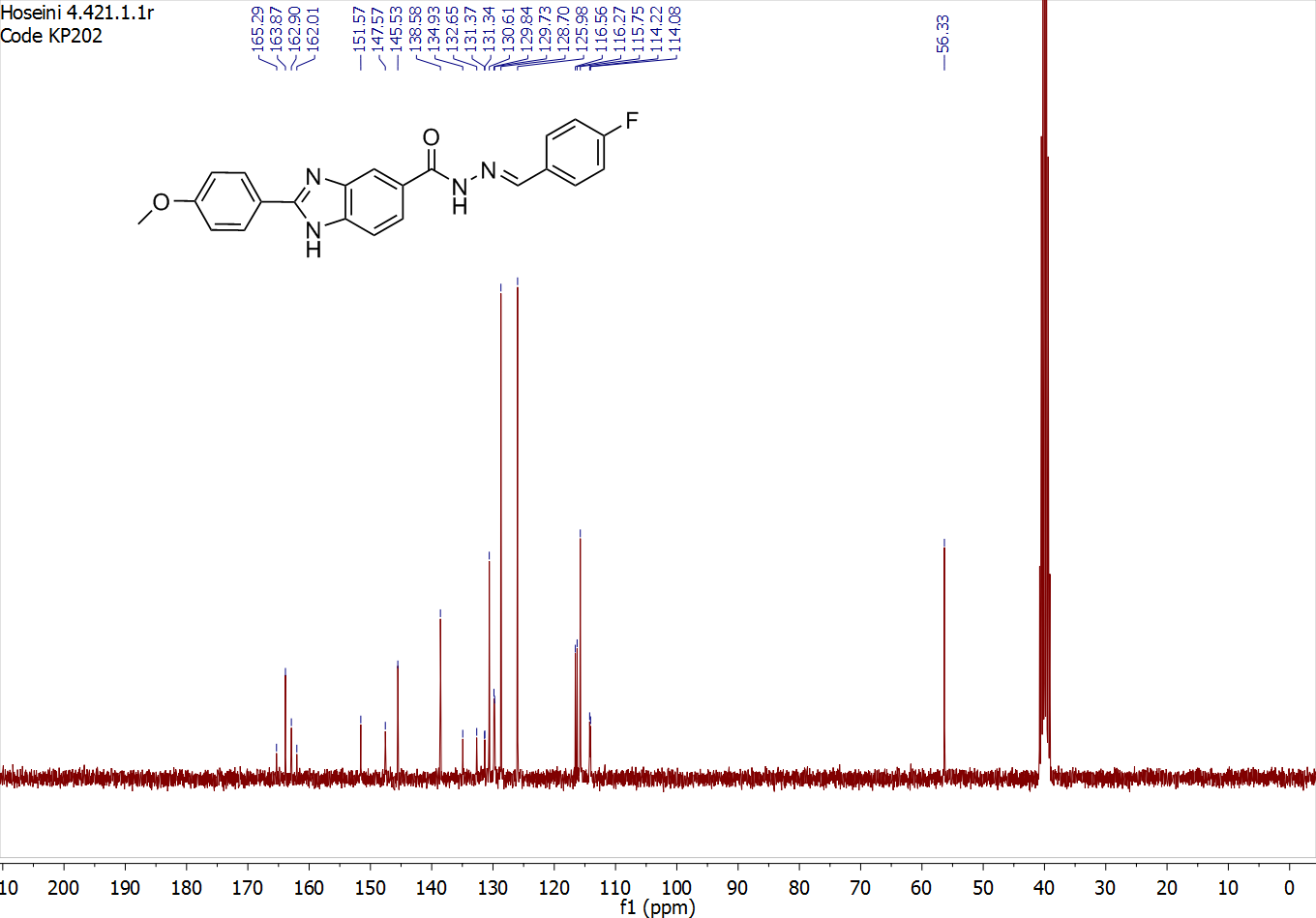


(*E*)-*N'*-(2-chlorobenzylidene)-2-(4-methoxyphenyl)-1*H*-benzo[d]imidazole-5-carbohydrazide (**8k**)


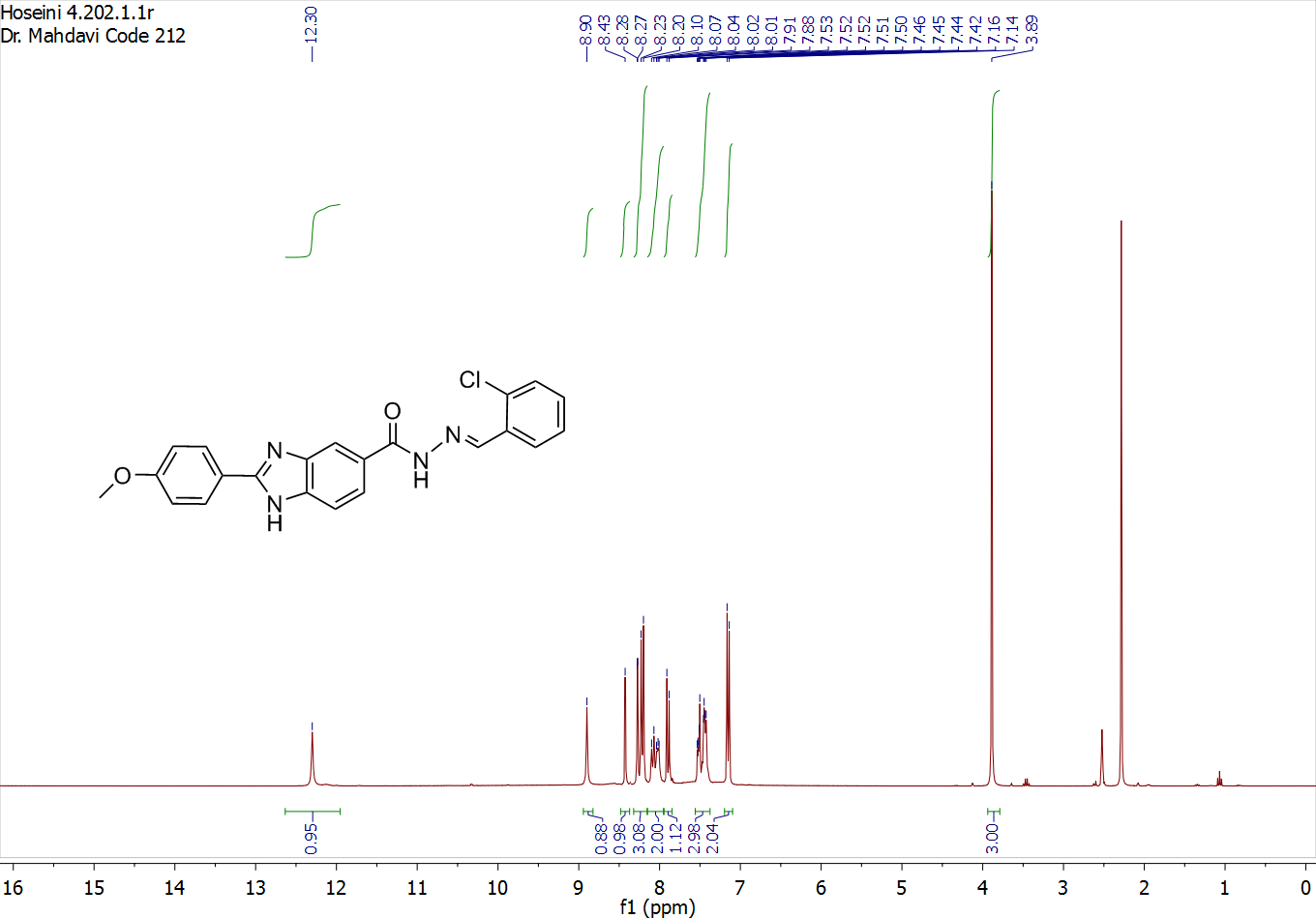


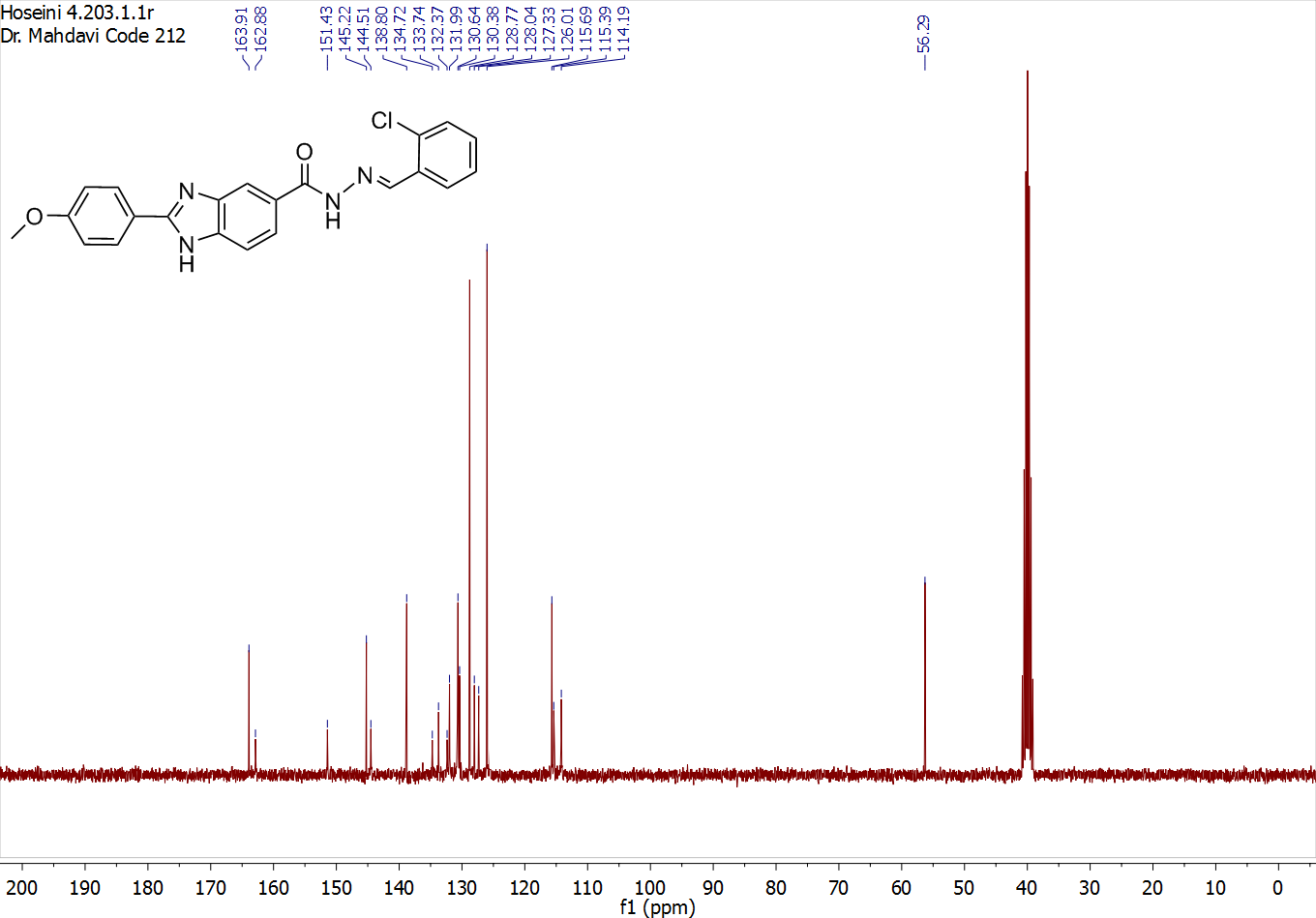


(*E*)-*N'*-(4-chlorobenzylidene)-2-(4-methoxyphenyl)-1*H*-benzo[d]imidazole-5-carbohydrazide (**8l**)


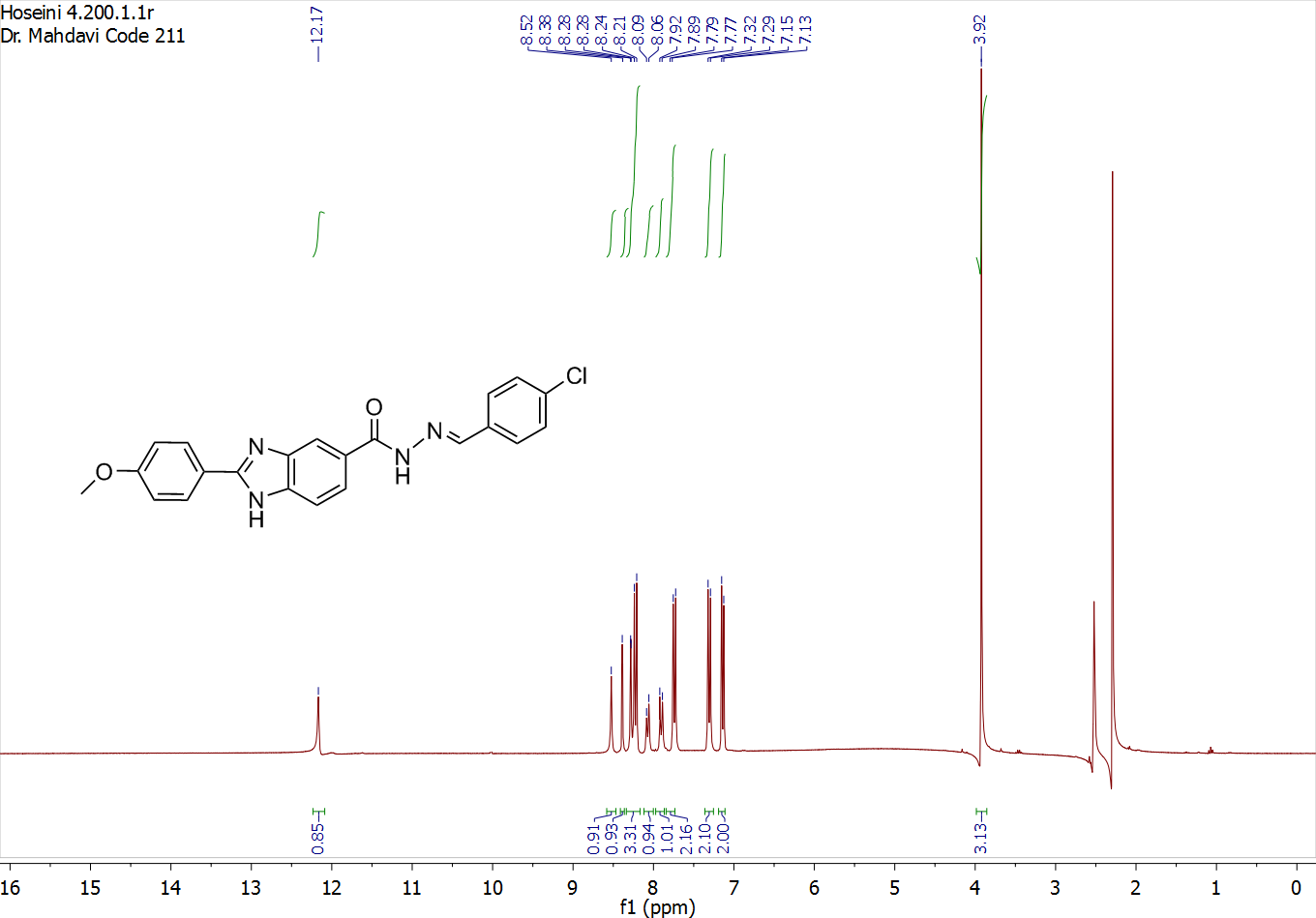


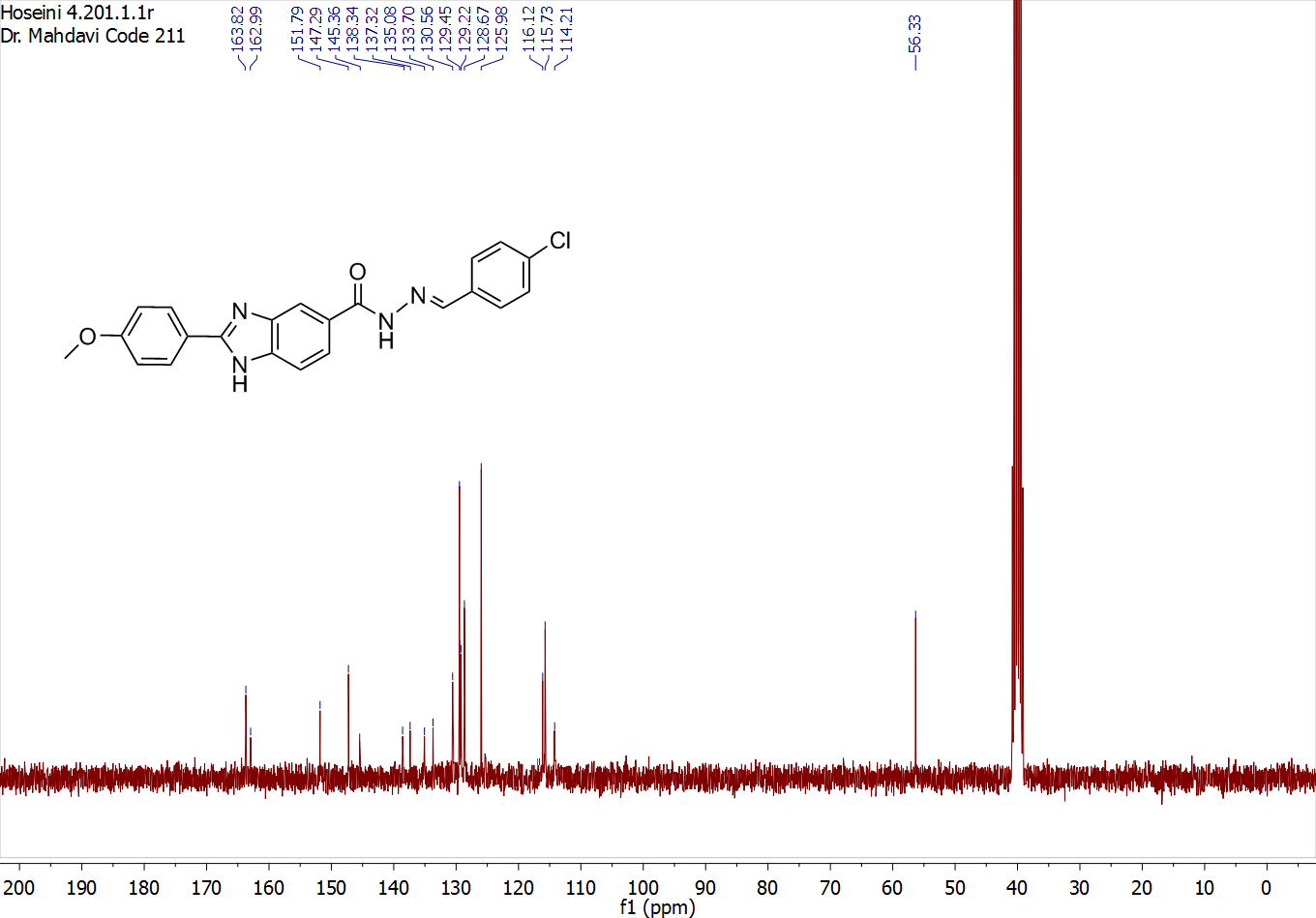


(*E*)-*N'*-(3-bromobenzylidene)-2-(4-methoxyphenyl)-1*H*-benzo[d]imidazole-5-carbohydrazide (**8m**)


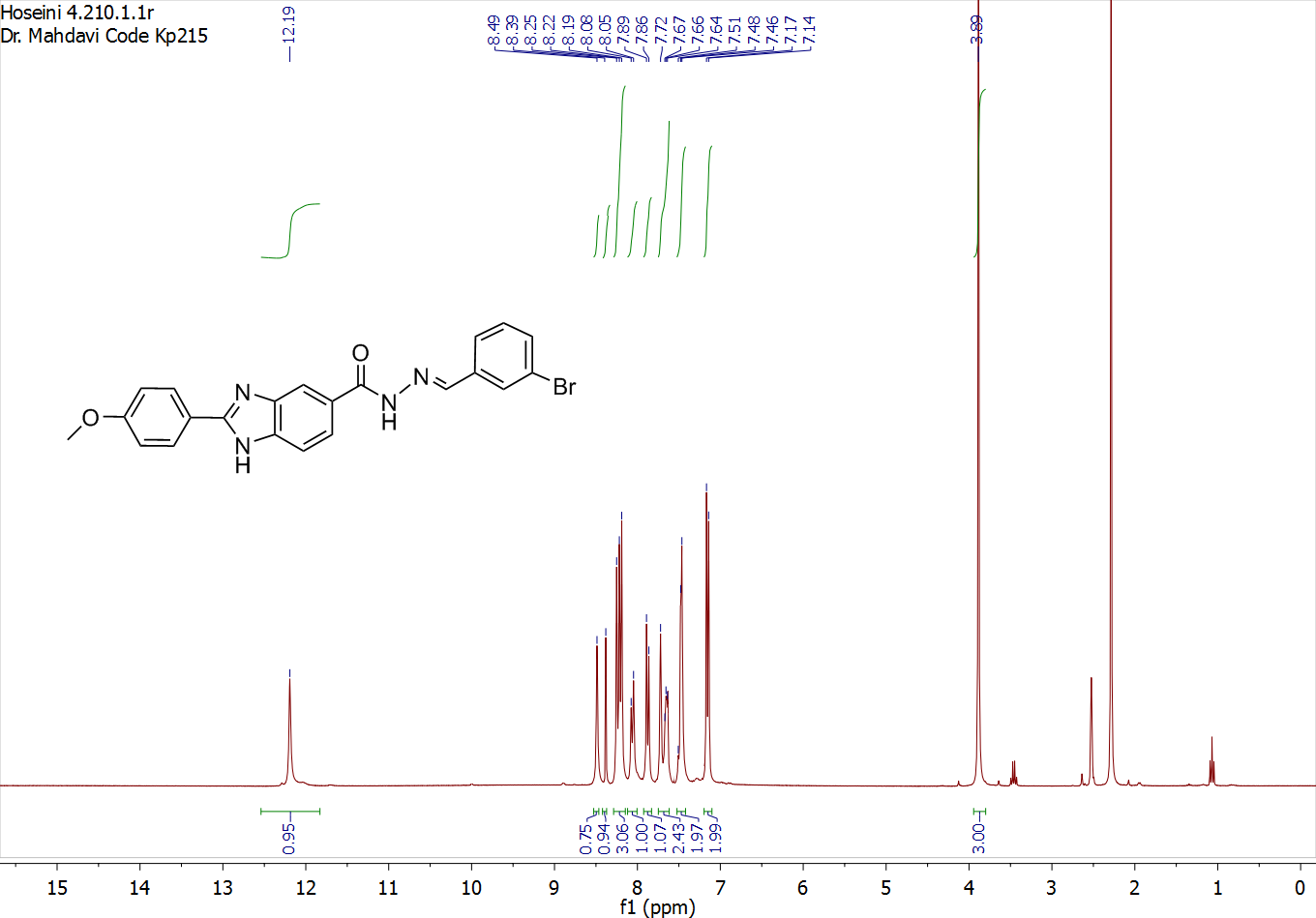


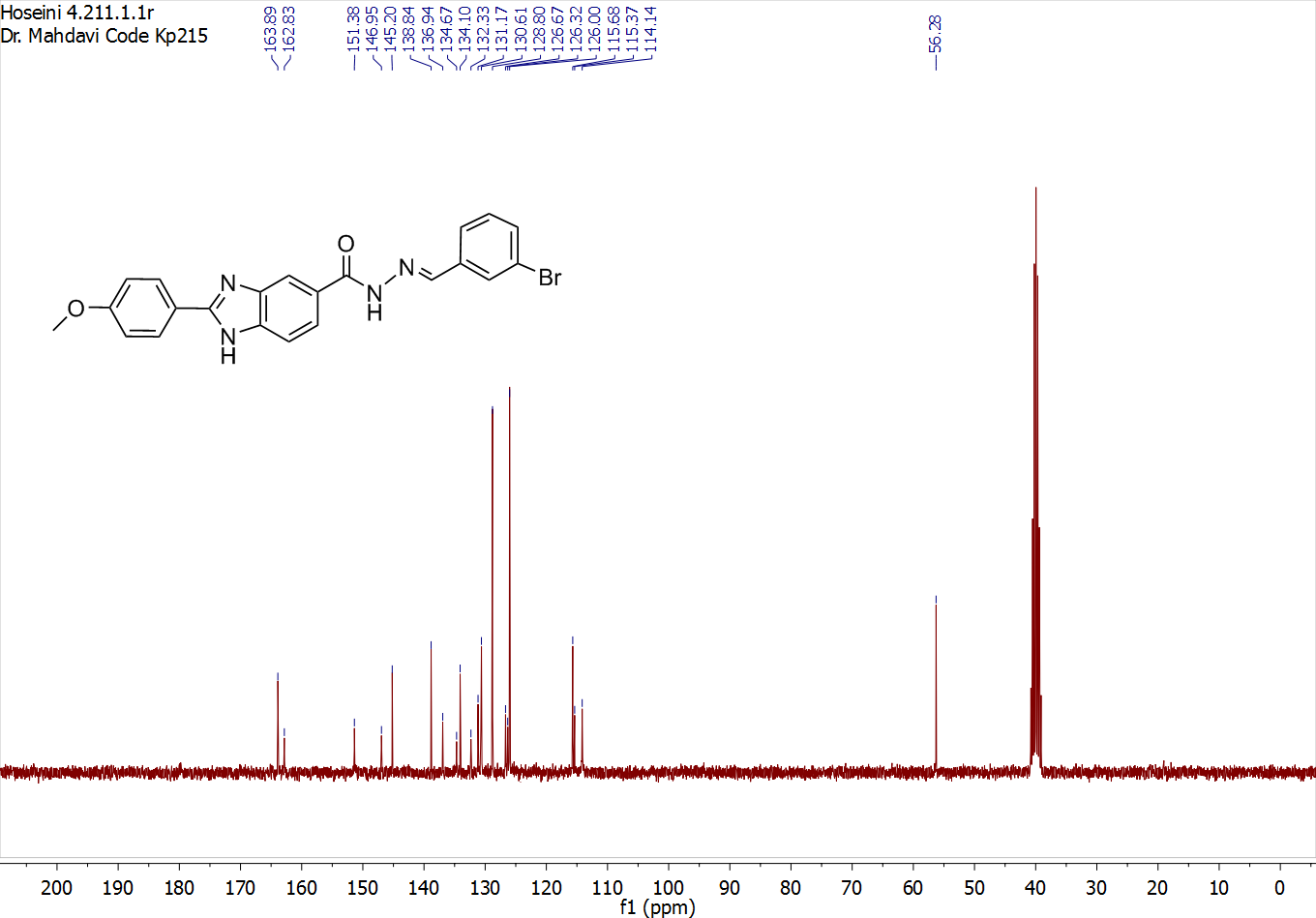


(*E*)-2-(4-methoxyphenyl)-*N'*-(4-nitrobenzylidene)-1*H*-benzo[d]imidazole-5-carbohydrazide (**8n**)


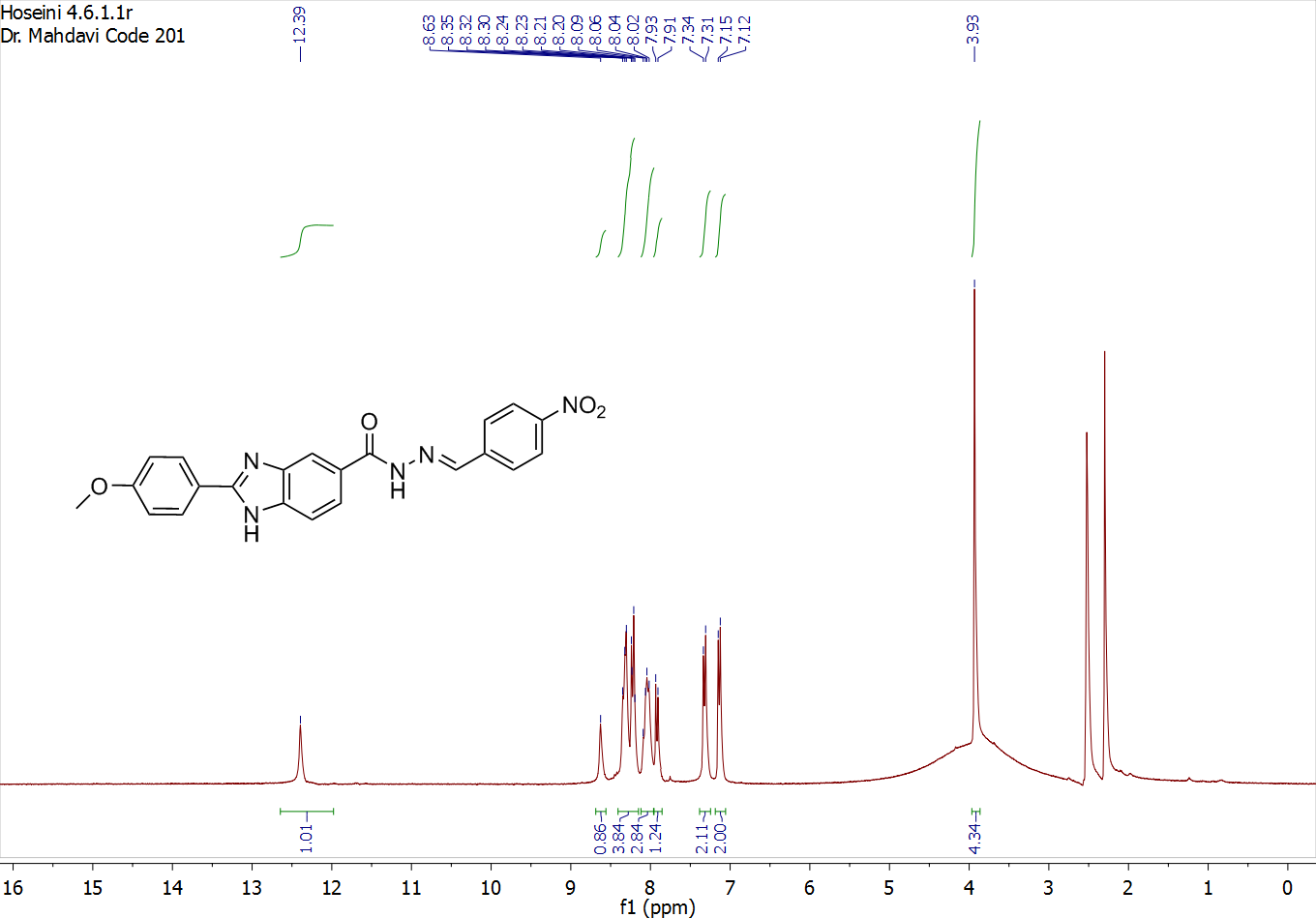


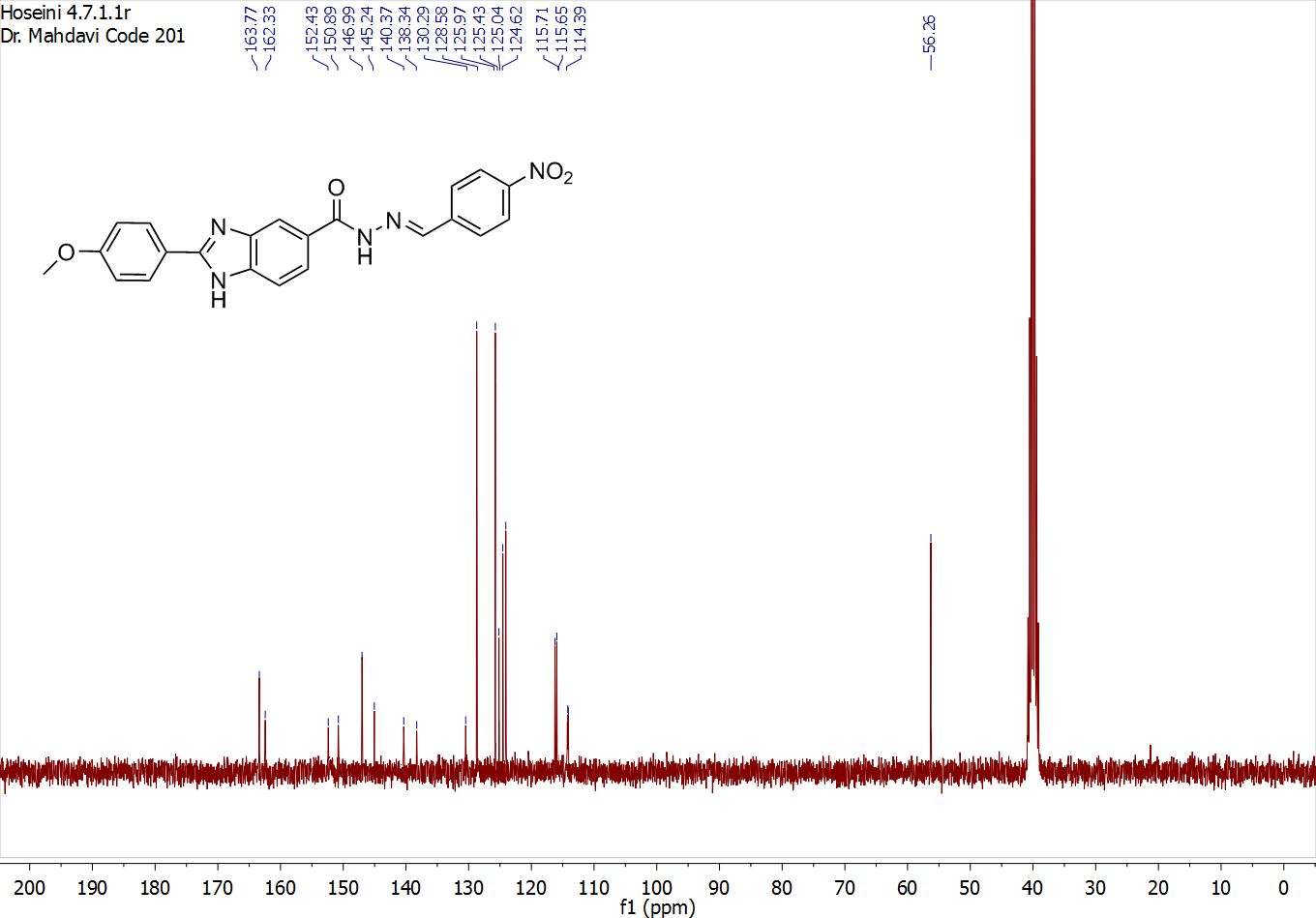


(*E*)-2-(4-methoxyphenyl)-*N'*-((6-nitrobenzo[d][1,3]dioxol-5-yl)methylene)-1*H*-benzo[d]imidazole-5-carbohydrazide (**8o**)


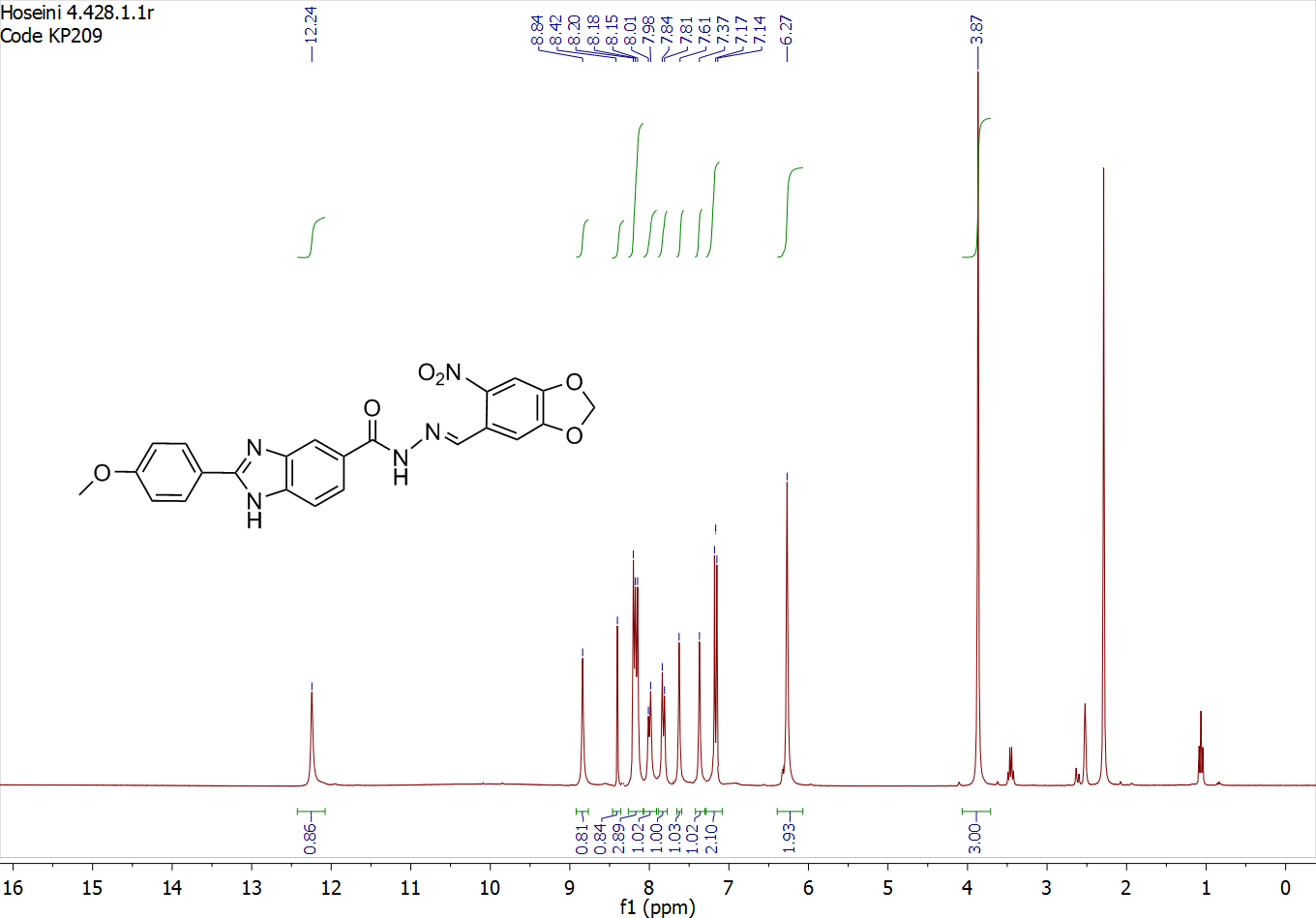


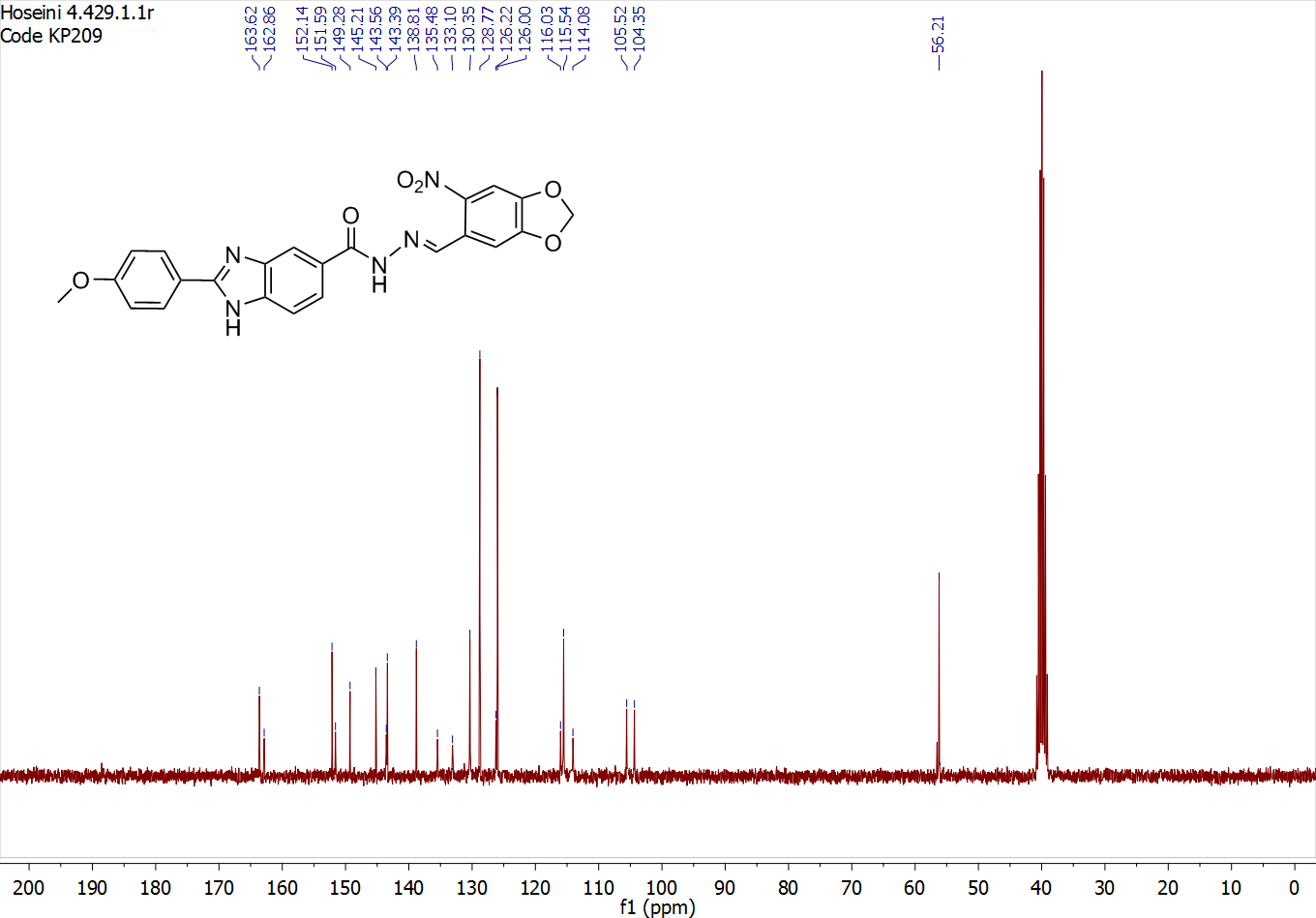


(*E*)-2-(4-methoxyphenyl)-*N'*-(thiophen-2-ylmethylene)-1*H*-benzo[d]imidazole-5-carbohydrazide (**8p**)


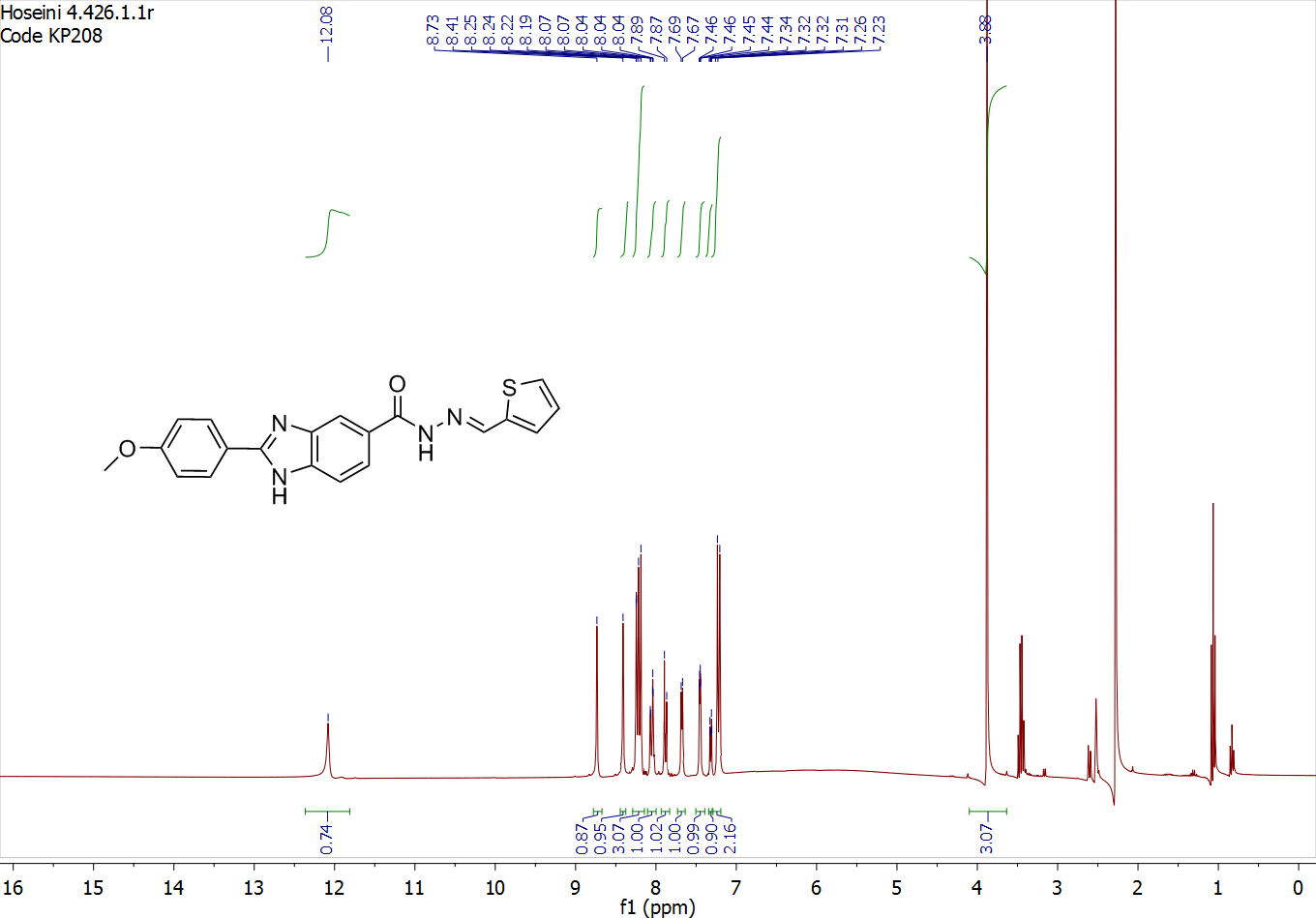


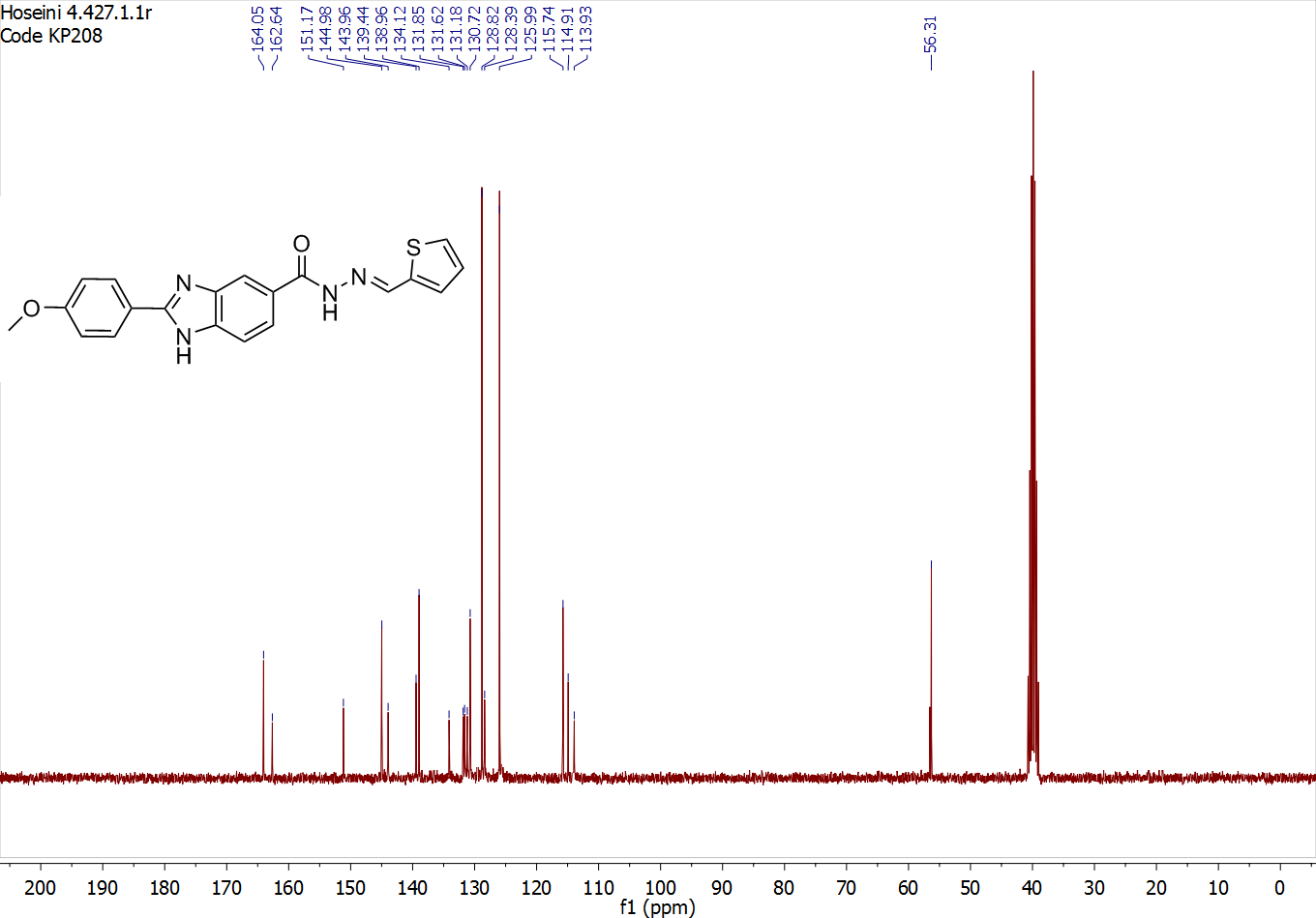

Supplement: Supplementary file 1 — Supplementary Information. [file 41598_2022_18896_MOESM1_ESM.docx]
